# Raised Activity of L-Type Calcium Channels Renders Neurons Prone to Form Paroxysmal Depolarization Shifts

**DOI:** 10.1007/s12017-013-8234-1

**Published:** 2013-05-22

**Authors:** Lena Rubi, Ulla Schandl, Michael Lagler, Petra Geier, Daniel Spies, Kuheli Das Gupta, Stefan Boehm, Helmut Kubista

**Affiliations:** Department of Neurophysiology and Neuropharmacology, Center of Physiology and Pharmacology, Medical University of Vienna, Waehringerstrasse 13a, 1090 Vienna, Austria

**Keywords:** Paroxysmal depolarization shift, Interictal spikes, L-type voltage-gated calcium channels, Acquired epilepsy, Neuropathogenesis

## Abstract

Neuronal L-type voltage-gated calcium channels (LTCCs) are involved in several physiological functions, but increased activity of LTCCs has been linked to pathology. Due to the coupling of LTCC-mediated Ca^2+^ influx to Ca^2+^-dependent conductances, such as K_Ca_ or non-specific cation channels, LTCCs act as important regulators of neuronal excitability. Augmentation of after-hyperpolarizations may be one mechanism that shows how elevated LTCC activity can lead to neurological malfunctions. However, little is known about other impacts on electrical discharge activity. We used pharmacological up-regulation of LTCCs to address this issue on primary rat hippocampal neurons. Potentiation of LTCCs with Bay K8644 enhanced excitatory postsynaptic potentials to various degrees and eventually resulted in paroxysmal depolarization shifts (PDS). Under conditions of disturbed Ca^2+^ homeostasis, PDS were evoked frequently upon LTCC potentiation. Exposing the neurons to oxidative stress using hydrogen peroxide also induced LTCC-dependent PDS. Hence, raising LTCC activity had unidirectional effects on brief electrical signals and increased the likeliness of epileptiform events. However, long-lasting seizure-like activity induced by various pharmacological means was affected by Bay K8644 in a bimodal manner, with increases in one group of neurons and decreases in another group. In each group, isradipine exerted the opposite effect. This suggests that therapeutic reduction in LTCC activity may have little beneficial or even adverse effects on long-lasting abnormal discharge activities. However, our data identify enhanced activity of LTCCs as one precipitating cause of PDS. Because evidence is continuously accumulating that PDS represent important elements in neuropathogenesis, LTCCs may provide valuable targets for neuroprophylactic therapy.

**Electronic supplementary material:**

The online version of this article (doi:10.1007/s12017-013-8234-1) contains supplementary material, which is available to authorized users.

## Introduction

L-type voltage-gated calcium channels (LTCCs) fulfill important neurological functions, for example as neuronal pacemakers, in synaptic plasticity and excitation-transcription coupling (Striessnig et al. 2006). However, elevated levels of LTCCs have been linked to pathology. LTCCs are up-regulated in aging neurons, and the incidence of several neurological diseases where LTCCs have been implicated, namely age-dependent memory deficits, Alzheimer’s disease (AD) and Parkinson’s disease (PD), increases with age (Moyer et al. 1992; Thibault et al. 2001, 2007; Veng and Browning 2002; Davare and Hell 2003; Veng et al. 2003; Chan et al. 2007, 2010; Sulzer and Schmitz 2007; Anekonda et al. 2011; Dursun et al. 2011; Ilijic et al. 2011; Kim and Rhim 2011). Furthermore, a gain of function mutation in Ca_v_1.2 has been linked to Timothy syndrome, which involves neurological dysfunction such as developmental delay and autism (Bidaud and Lory 2011). There is also evidence that hyperactive LTCCs play a role in epileptic disorders. For example, in a subpopulation of neurons of the spontaneously epileptic rat (SER), the group of Masashi Sasa found by comparison of current–voltage relation curves that voltage-gated calcium currents are activated at considerably less depolarized voltages than in neurons of non-epileptic control rats (Yan et al. 2007). Indirect evidence from earlier studies of this group indicates that the channel responsible for this alteration in calcium current is an LTCC (e.g., Amano et al. 2001a and 2004). Furthermore, in neurons of the seizure prone gerbil, protein levels of Ca_v_1.3 were found to be increased (Park et al. 2003; Kang et al. 2004). Similar to the above-named neurological dysfunctions, the incidence of epilepsies also increases with age (Werhahn 2009).

LTCCs have long been suggested to act as important regulators of neuronal excitability, and their coupling to Ca^2+^-dependent conductances is known to play a crucial role in shaping neuronal discharge patterns (Moyer et al. 1992; Morisset and Nagy 1999). Enhanced LTCC-mediated after-hyperpolarizations were suggested to be causally linked to age-related cognitive impairment (see for example Gamelli et al. 2011). However, in a previous study (Geier et al. 2011), we showed by potentiation of LTCCs that these voltage-gated calcium channels have both excitatory and inhibitory coupling modes in neurons of rat hippocampus, and both coupling modes can operate in a given neuron. Hence, it remained unknown whether, in which direction, and to what extent pathologically enhanced LTCC activities may affect the discharge properties of neurons. To address these questions, we performed patch-clamp recordings from various types of hippocampal neurons in primary culture and studied the effects of pharmacological up-regulation of LTCCs (denoted as “LTCC↑” in the following) in current-clamp recordings of neuronal activity.

## Materials and Methods

### Primary Cell Culture of Hippocampal Neurons

Hippocampi were dissected from neonatal Sprague–Dawley rats which had been killed by decapitation, and primary cultures of hippocampal neurons were prepared in the same manner as described previously (Geier et al. 2011). Hence, all experiments were performed ex vivo.

### Electrophysiology/Measurements of Neuronal Activity and Seizure-like Activity Using Current clamp

Recordings of membrane voltage were performed using a Multiclamp 700B amplifier (Axon Instruments) in the current-clamp mode. Signals were low-pass filtered at 10 kHz and digitized with a Digidata 1440A digitizer (Molecular Devices, Sunnyvale, CA, USA) at a sampling rate of 20 kHz. Patch pipettes were made of borosilicate capillaries (GB150-8P, Science Products, Hofheim, Germany) with a Sutter P97 horizontal puller (Sutter Instrument Company, Novato, CA, USA). Tip resistances lay between 3.5 and 5 MΩ. Pipette solutions contained (in mM) 120 potassium gluconate, 1.5 sodium gluconate, 3.5 NaCl, 1.5 CaCl_2_, 0.25 MgCl_2_, 10 HEPES, 10 glucose and 5 EGTA. pH was adjusted to 7.3 by KOH. For perforated patch recordings, 500 μg/ml amphotericin B (from Streptomyces sp., compound purchased from Sigma-Aldrich, Vienna, Austria) was added to the pipette solution. Experiments were started only after the series resistance had dropped to the lowest achievable level (e.g., between 20 and 30 MΩ), which usually occurred within 15–30 min. To assure that only viable cells were used, the following inclusion criteria had to be met: a membrane voltage of at least −50 mV and the capability of generating overshooting action potentials, which was always tested prior to the recordings. Typically, the neurons had a membrane resting potential of slightly less negative than −70 mV (−67.3 ± 6.3 mV, mean ± SD, as determined from 45 neurons used in this study). Experiments were performed at room temperature, and cells were superfused continuously with standard external solution containing (in mM) 140 NaCl, 3 KCl, 2 CaCl_2_, 2 MgCl_2_, 10 HEPES, 20 glucose (pH was adjusted to 7.4 by NaOH). LTCC activity was modulated by application of the dihydropyridines isradipine (“isra,” LTCC antagonist) and Bay K8644 (“BayK,” LTCC agonist), both at 3 μM in all experiments.

The low Mg^2+^ model of epilepsy represents one of the most widely used in vitro models of epilepsy and is thought to rely on the relief of the physiological Mg^2+^ block of NMDA receptors (Stanton et al. 1987; Solger et al. 2005). In this study, SLA was evoked by 2 min of superfusion with nominally Mg^2+^-free buffer, and recordings were made under control conditions (DMSO only) and in the presence of BayK and isradipine, respectively, both at 3 μM. SLA typically either appeared instantaneously or at least within 30 s. Before LTCC modulators were tested, at least two control SLAs were recorded. Only the second control recording was used for comparisons. Between low-Mg^2+^ applications, cells were allowed to recover for 5 min by superfusing with standard external solution. Normal electrical activity re-appeared typically within the first minute of Mg^2+^ readdition.

### Electrophysiology/LTCC Current Measurements Using Voltage Clamp

Recording of voltage-gated calcium currents from fully differentiated neurons represents a challenging undertaking, in particular because of space-clamp problems. Moreover, LTCC currents are prone to show substantial run down, a problem that can be alleviated by addition of an ATP-regenerating system in whole cell patch–clamp experiments (see for example Bruehl et al. 2000) or by using the perforated patch method. We opted for the later approach, first of all because compounds in the ATP-regenerating system may not only dampen run down but may also alter endogenous LTCC activities, and secondly because current-clamp recordings were performed in perforated patch method, and we aimed at testing LTCC availability under closely matching conditions. Unfortunately, perforated patch recordings typically come with high access resistance, a situation that impairs voltage control. To overcome this problem, we used an approach previously employed by other authors (e.g., Deak et al. 2000) in which ramp depolarizations are applied to the neurons. In these experiments, the pipette solution contained (in mM) 120 CsCl, 10 HEPES, 5 EGTA, 1.5 CaCl_2_, 0.25 MgCl_2_ and 5 NaCl adjusted to pH 7.30 using 5 M CsOH. And the standard external solution here contained (in mM) 120 NaCl, 20 TEA-Cl, 3 KCl, 2 CaCl_2_, 2 MgCl_2_, 20 Glucose, 10 HEPES and 0.0005 tetrodotoxin (TTX) and was adjusted to pH 7.4 with NaOH. 500 μg/ml amphotericin B (from *Streptomyces* sp., compound purchased from Sigma-Aldrich, Vienna, Austria) was added to the pipette solution just before seal formation.

### Drugs

4-Aminopyridine, BayK, caffeine, dimethyl sulfoxide (DMSO), H_2_O_2_, isradipine and bulk chemicals were purchased from Sigma-Aldrich (Vienna, Austria), and XE 991 dihydrochloride from Tocris Bioscience (Bristol, UK). Since some of these drugs were dissolved in DMSO, the concentration of this solvent was kept constant at 0.3 % in all solutions. Control solution contained 0.3 % DMSO only, whereas DMSO-soluble compounds were diluted from concentrated stock solutions so as to obtain the same final concentration of DMSO.

Dihydropyridines have been widely used as LTCC modulators. However, dihydropyridine-type LTCC inhibitors may act on other than calcium channels (see for example Perez-Reyes et al. 2009). Importantly, no non-specific activating effect has been found for the dihydropyridine-type LTCC agonist BayK. Hence, we identified LTCC effects as being inhibited by isradipine and augmented by BayK (Geier et al. 2011). Here, the responses that were elicited (or augmented) by BayK and inhibited by isradipine can thus be *bona fide* considered as LTCC-dependent events.

### Data Analysis and Statistics

For identification of spontaneously occurring excitatory postsynaptic potentials, a threshold search operation (“event detection”) was performed on typically 5-min-long recordings made under each experimental condition using Clampfit 10.2, which is part of the pCLAMP 10 electrophysiology data acquisition and analysis software package (Molecular Devices, Sunnyvale, CA, USA). To detect and analyze the events for peak voltage and area below the curve, the threshold was set to 15 mV and re-arm to 5 mV above baseline. Later on in this study, for identification of paroxysmal depolarization shifts (PDS), we adapted these parameters to specifically scan our recordings for time periods which the neurons spent at a considerably depolarized potential. To set the parameters appropriately, we went back to the earliest description of these epileptiform events, where they were recorded both by means of extracellular and intracellular methods. Matsumoto and Ajmone Marsan (1964a) described PDS as a “membrane positive shift, up to 30 mV or, occasionally more” with durations typically in the range from 40 up to 400 ms, sometimes even longer. These authors also noted that PDS start with an initial action potential which is followed by a progressive inactivation of spike generation in the course of the PDS so that only small oscillations remain riding on top of the depolarization shift. Similar observations were made in other seminal work in the field (e.g., Moraidis et al. 1991). Hence, search parameters were chosen to identify depolarizing events in our recordings which exceeded a size of 20 mV for a prolonged period of time and showed (at least initially) spike firing. An illustration of the event detection routine used for this purpose is provided in the electronic supplementary material (Online Resource 1). The size of events identified was calculated by measuring the area between the recorded trace and a virtual baseline set at 20 mV above the membrane resting potential. Statistical analysis of the event areas (mV·ms) for each experimental condition and comparisons thereof was carried out using Graphpad Prism 5.03 (see below). Finally, the number of events exceeding a certain area level, typically 1,000 mV·ms (“PDS1000”), but in some cases also 500 mV·ms (“PDS500”), was determined. A similar (yet by eye-made) evaluation of PDS from various publications yielded areas typically larger than 1,000 mV·ms (see for example Witte et al. 1987), in some cases even several thousand mV·ms (see for example Schiller 2002), but PDS with areas between 500 and 1,000 mV·ms were also encountered (see for example Ben-Ari et al. 1989). Here, for each experimental condition, area analysis was performed on 120-s-long recordings; hence, the number of PDS (“# of PDS”) given in the graphs also always refers to a 2-min time frame. Seizure-like activity (SLA) was also quantified using Clampfit 10.2 software by measuring the area between the voltage trace and a baseline corresponding to the average resting membrane potential of the neuron prior to the onset of SLA.

GraphPad Prism version 5.03 was used for preparation of the graphs (all data are represented as mean ± SEM, unless otherwise stated) and for all other statistical testing. Wilcoxon matched-pairs signed rank test, Kruskal–Wallis one-way ANOVA with Dunn’s post hoc test and repeated measures ANOVA with Dunnett’s or Tukey’s multiple comparison test were selected as required by the type of the data (see figure legends). For selection of the statistical test, normality tests were performed using D’Agostino and Pearson omnibus normality test or Kolmogorov–Smirnov test, depending on the sample sizes.

## Results

### Effect of LTCC↑ on Sub- and Supra-threshold EPSPs

**Fig. 1:**
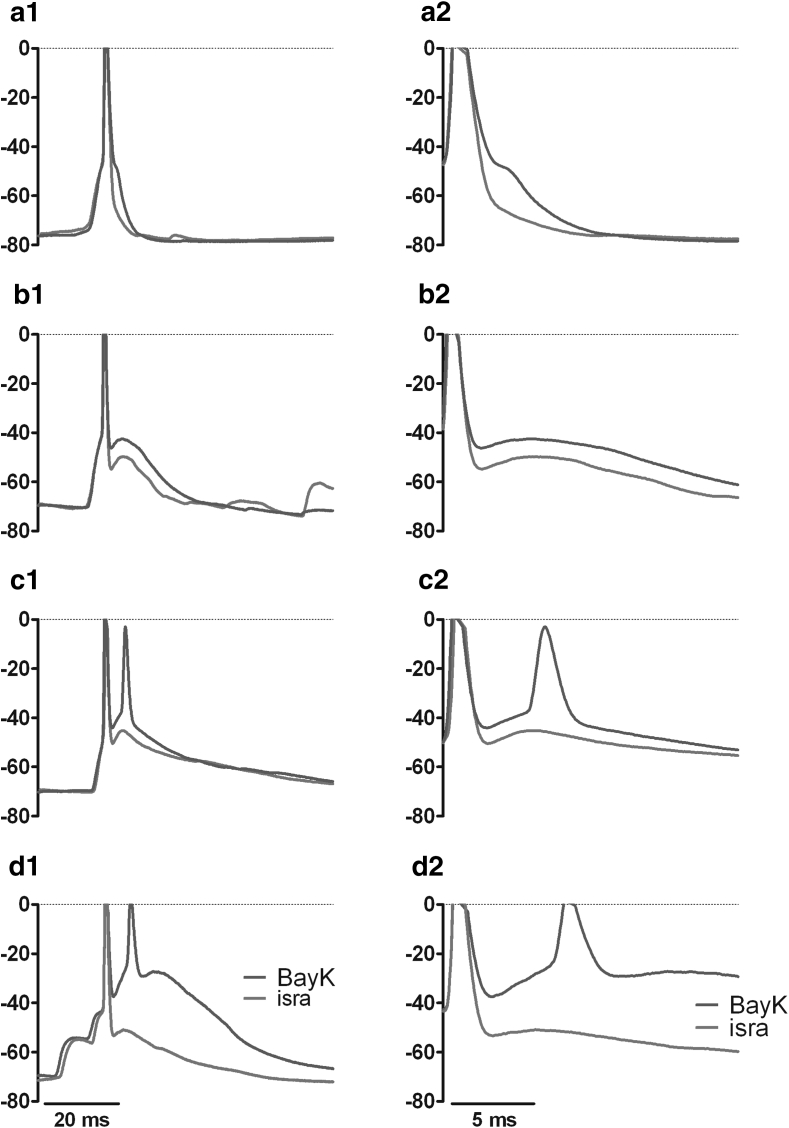
Effect of LTCC activity on EPSPs-1. Pharmacological potentiation of LTCCs unequivocally augments supra-threshold EPSPs, albeit at varying degrees among hippocampal neurons. The effect range of pharmacological up-regulation of LTCCs on spontaneously occurring supra-threshold EPSPs is illustrated in overlays of traces recorded in the presence of BayK (*green traces*) and isradipine (*red traces*), respectively, in ascending sequence from **a** to **d**. Traces were aligned with respect to the first spike in the EPSP. Overlays on the *left* show the entire EPSPs (a1–d1); the overlays on the *right* show the postspike part of the same EPSPs on an expanded time scale (a2–d2). For a better visualization of the non-overshooting part of the events, the recordings in this and all subsequent figures are shown truncated at 0 mV. *Y-*axes units in this and all subsequent figures are in mV (Color figure online)

**Fig. 2:**
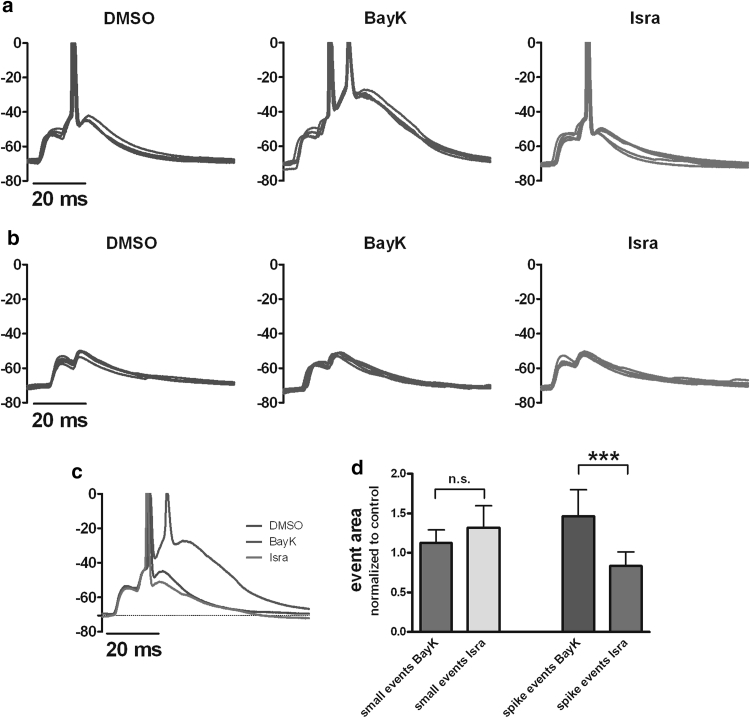
Effect of LTCC activity on EPSPs-2. Pharmacological potentiation of LTCCs augments (short) super-threshold synaptic potentials (“spike events,” **a**) and promotes the formation of depolarization shifts (see middle traces in **a**), but at the same time leaves sub-threshold EPSPs (“small events,” **b**) unaltered. Isradipine reverses the effect of BayK. Each graph shows an overlay of 5 arbitrarily chosen EPSPs recorded in DMSO (*dark blue traces*), BayK (*green traces*) and isradipine (*red traces*). **c** Overlay of representative traces from this experiment recorded under the three experimental conditions. **d** Statistical comparisons of small event and spike event data, respectively, from a total of 12 experiments identical to the one illustrated in **a**–**c** (see main text for details). n.s. indicates a lack of statistical significance, ***P value <0.001 (Color figure online)

To start our investigations on the least complex neuronal signals, we tested the effect of LTCC modulation on spontaneously occurring excitatory postsynaptic potentials (EPSPs). To facilitate the detection of individual EPSPs, hippocampal neurons were slightly hyperpolarized by injection of a negative holding current (−10 to −100 pA). Five-min-long recordings were made under control conditions (with DMSO), in the presence of 3 μM BayK and after exchange of BayK with 3 μM isradipine (*n* = 12). Potentiation of LTCCs with BayK in no case reduced the spontaneously occurring EPSPs but always augmented them, albeit to varying degrees. Figure 1 illustrates in overlays of original traces recorded in the presence of BayK and isradipine the maximum range in which changes in EPSPs occurred when LTCCs were potentiated (BayK, green traces) or blocked (isradipine, red traces). EPSPs were quantified as explained in “Materials and Methods” section with respect to peak voltage (mV) and area below the curve (mV·ms). Peak voltage data were used to group the events according to whether they remained below the threshold for action potential firing (“small events,” not exceeding −50 mV) or whether the spontaneous synaptic potentials led to action potential discharge (“spike events”). From the last 100 s of recording under each experimental condition, 5 identified events were arbitrarily chosen and displayed in overlays. This is illustrated for a neuron with a pronounced effect of BayK on spike events in Fig. 2a. Upon exchange of BayK for isradipine, events were reduced to at least the control level in the presence of isradipine (Fig. 2a, right traces). In the same neuron, comparison of small event traces did not reveal any obvious effect of LTCC modulation (Fig. 2b). Statistical comparison (one-way ANOVA with Tukey’s posttest) of all events recorded within the 5-min test periods in this neuron showed that whereas small events showed no significant difference under the three experimental conditions, spike events were enhanced with high statistical significance (*P* value <0.001) in the presence of BayK 2.1-fold and were reduced with low statistical significance upon application of isradipine (*P* value <0.05) to 74 % of the control value in this particular neuron (data not shown). An overlay of averaged traces illustrates this result (Fig. 2c). To confirm this observation, separate analysis for small and spike events was performed for all 12 neurons tested. To enable statistical comparisons of pooled data, event areas were normalized to control (DMSO). Data from these experiments are summarized in the graph shown in Fig. 2d. As indicated, statistical analysis showed that small events recorded in BayK did not differ from small events occurring in the presence of isradipine (*P* value = 0.62, Wilcoxon matched-pairs signed rank test). In contrast, there was a highly significant difference between areas of spike events recorded in the presence of BayK and isradipine, respectively (*P* value of the statistical comparison was 0.0002, Wilcoxon matched-pairs signed rank test). Overall, the median of event areas rose to 1.46 ± 0.34 in the presence of BayK and fell to 0.83 ± 0.18 in the presence of isradipine (Fig. 2d, right bars).

### Capability of LTCC↑ to Induce PDS

**Fig. 3:**
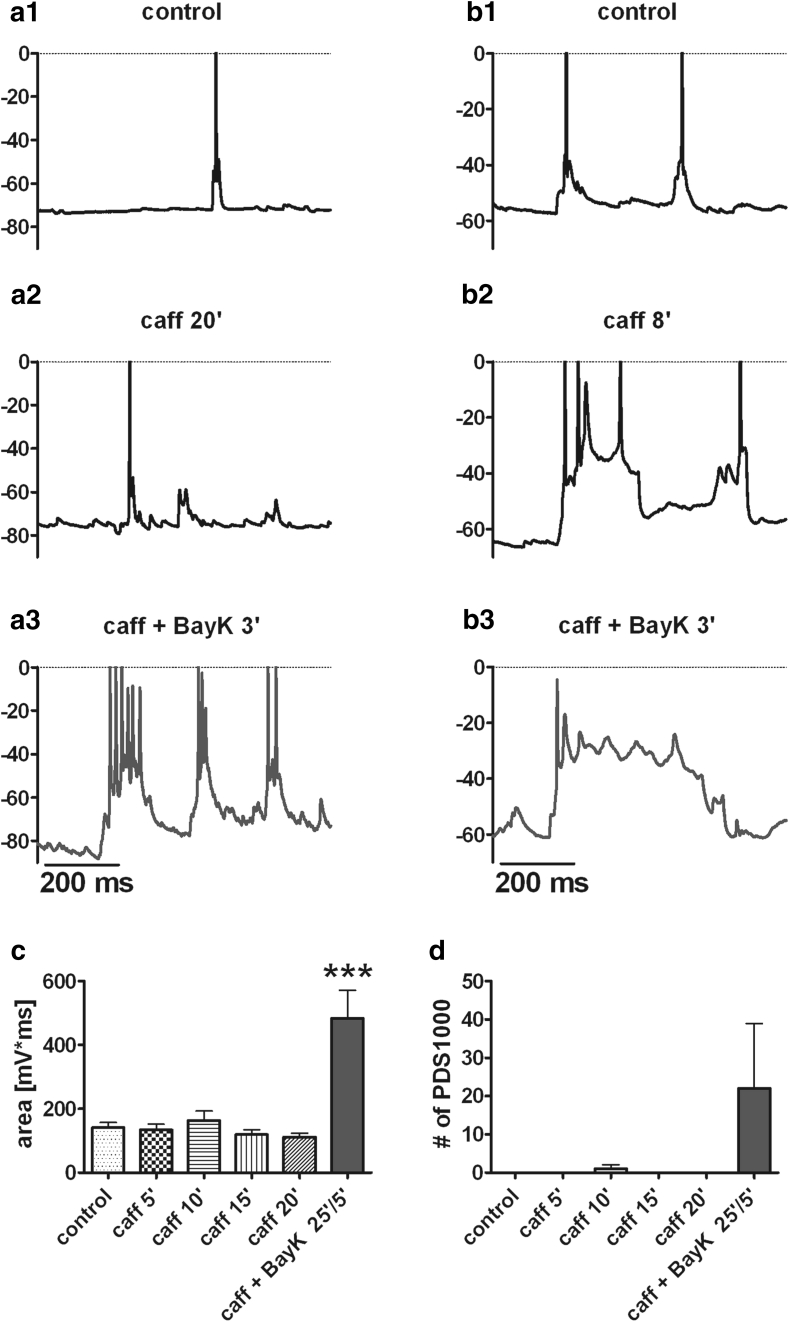
Caffeine is inefficient on its own to induce PDS but readily does so upon co-administration of BayK. **a**, **b** As illustrated by original traces, caffeine (1 mM) in all (**a**1, **a**2) but one (**b**1, **b**2) out of 11 neurons failed to induce PDS within 20 min. However, PDS were readily observed after addition of 3 μM BayK (**a**3, **b**3). Indicated on top in each graph is the time at which the trace shown was recorded, for example the trace in **a**2 was recorded 20 min after switching to caffeine-containing saline. **c**, **d** Analysis of this set of experiments according to event area (mV·ms) of depolarizing events and number of depolarization shifts with an area exceeding 1,000 mV·ms (“PDS1000,” see “Materials and Methods” section for details). Data were collected from 6 experiments where BayK showed a prominent effect with respect to PDS formation by evaluating 2-min time frames, starting 2 min prior to and ending at the time indicated on the *x-*axes; for example the caff 5’ data point represents the events that occurred between 3 and 5 min after switching to caffeine-containing saline. No significant difference (n.s.) in event area was found between control data and events recorded in the presence of caffeine. However, event area significantly increased upon subsequent application of BayK (**c**, ***P < 0.001, repeated measures ANOVA followed by Dunnett’s multiple comparison test). This increase in average event area was paralleled by the appearance of PDS1000 events (**d**)

The most pronounced enhancement of EPSPs (e.g., Fig. 2a) led to voltage responses that were reminiscent of PDS, pathologically elevated depolarization waveforms seen for example in animal models of acquired epilepsies (prior to the onset of the first seizure) but also recognized as the cellular correlate of interictal spikes (IIS) (Matsumoto and Ajmone Marsan 1964a, b, c; De Curtis and Avanzini 2001). To date, the etiology of PDS formation is far from being understood. Earlier studies using verapamil and some of its derivates suggested that LTCCs may contribute to PDS (Moraidis et al. 1991; Schiller 2002), yet how exactly LTCCs may come into play in these abnormal electrical events remained obscure. It has been shown by the seminal work of E. Speckmann’s group (University of Münster, Germany) that in hippocampal slices PDS can be induced by application of millimolar caffeine (e.g., Moraidis et al. 1991). Hence, we were interested in how caffeine-induced PDS might be affected by pharmacological up- and down-regulation of LTCCs. Interestingly, in contrast to earlier studies on hippocampal networks, in our hands 1 mM caffeine alone within 20 min in all but one out of 11 neurons failed to generate PDS-like depolarizing events (Fig. 3). In this particular neuron, the depolarization shift was further enhanced by BayK, giving rise to a particularly pronounced PDS (Fig. 3b1–b3). Of the other 10 neurons, addition of BayK (3 μM) in the continuous presence of caffeine evoked depolarizing shifts in 5 cases. Hence, all together 6 out of 11 neurons tested generated PDS upon pharmacological potentiation of LTCCs (Fig. 3a3, b3). The inability of caffeine on its own to evoke PDS in these dihydropyridine-sensitive neurons is illustrated in Fig. 3c by means of area analysis and in Fig. 3d by the determination of the number of depolarization shifts which exceeded an area of 1,000 mV·ms within 2 min of observation (“PDS1000,” see “Materials and Methods” section and Online Resource 1 for a detailed description of the analysis).

**Fig. 4:**
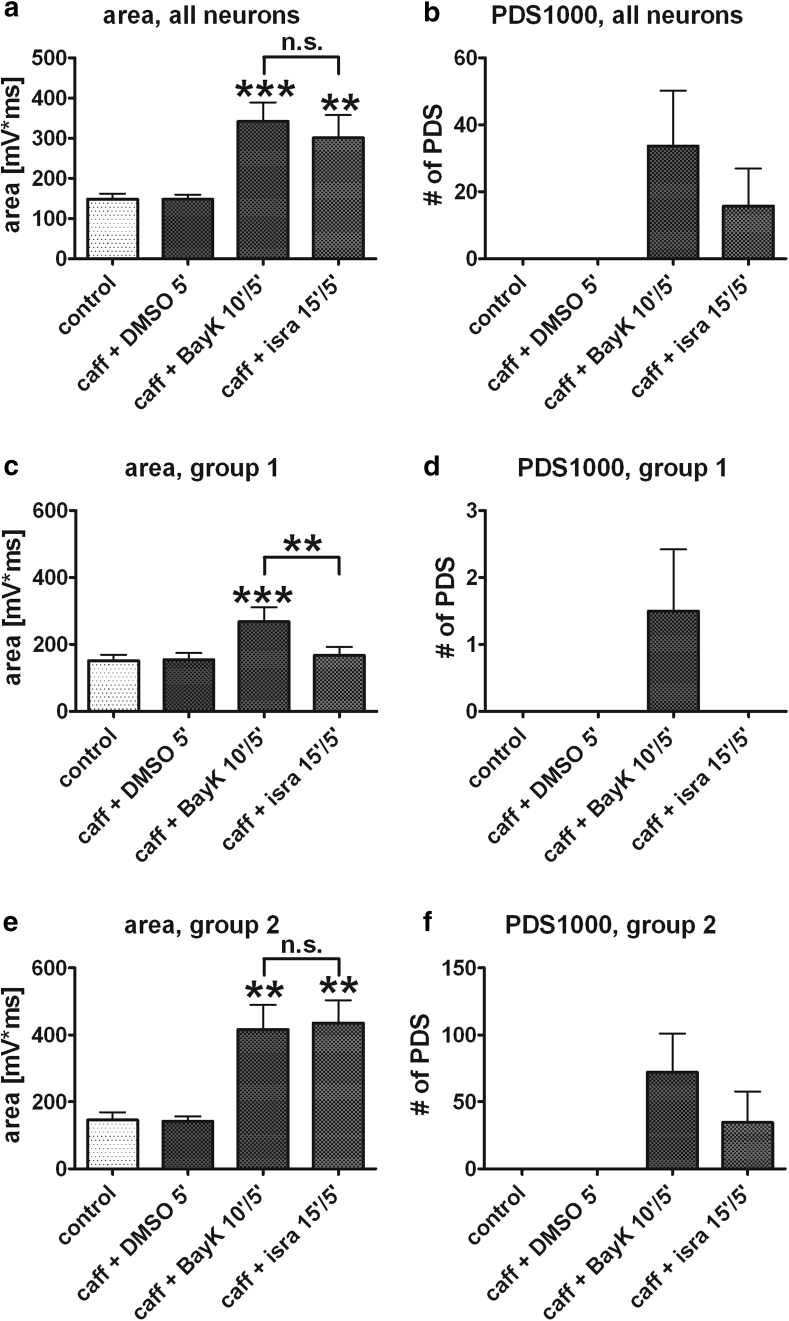
Reversible and irreversible induction of PDS. **a**, **b** Collectively, isradipine proved ineffective in suppressing BayK-induced PDS, as shown in **a** for event area and in **b** for PDS1000 (*n* = 10). **c**–**f** However, closer inspection of the data revealed the existence of two populations of neurons: one where PDS induction by BayK was moderate (group 1, *n* = 5) and fully inhibited after addition of isradipine (**c**, **d**) and another one (group 2, *n* = 5) where BayK led to pronounced appearance of PDS, an effect that was hardly reduced after exchange of BayK for isradipine (**e**, **f**). *** and ** above the *error bars* indicate *P* ≤ 0.001 and *P* ≤ 0.01, respectively, for statistical comparison of the marked data versus control using repeated measures ANOVA followed by Dunnett’s multiple comparison test. In a further comparison of all columns using repeated measures ANOVA with Tukey’s multiple comparison test, statistical difference was also examined between the caffeine + BayK and the caffeine + isradipine data (*horizontal brackets*): n.s. indicates a lack of statistical difference and **significant difference with *P* ≤ 0.01

We moved on to study BayK-induced PDS (in the presence of caffeine) in more detail. Out of 16 neurons investigated, 10 neurons could be used for further analysis of LTCC-mediated PDS for the following reason: in 5 of the 16 neurons, no effect on discharge activities resembling PDS could be detected by eye, although close inspection of the recordings revealed augmentation of EPSPs (not shown). In one neuron, long-lasting plateau potentials (with event durations exceeding 1 s) were evoked, and therefore this neuron was also omitted from evaluation. In the 10 remaining neurons in which BayK led to clearly discernible PDS-like events, the effect of exchange of BayK for isradipine was studied. Unexpectedly, isradipine did not eliminate PDS and the increase in event area remained statistically unaltered as compared to the BayK results (Fig. 4a), although there was a tendency toward a reduced number of PDS1000 (Fig. 4b). However, closer inspection of these data showed that they could be divided in two groups: in one group (group 1, 5 neurons), the effect of BayK was moderate (1.7-fold increase in event area, only 1–2 PDS1000 evoked within a 2-min time frame), but was entirely reversible upon administration of isradipine (Fig. 4c, d). In the other group (group 2, also 5 neurons), a pronounced PDS-inducing effect was noted with BayK (2.9-fold increase in event area, frequency of PDS1000 reaching 0.6 Hz on average), but this effect could not be reversed by administration of isradipine (Fig. 4e, f). Hence, isradipine appeared only capable of reversing moderate induction of PDS-like events initiated by preceding LTCC potentiation.

**Fig. 5:**
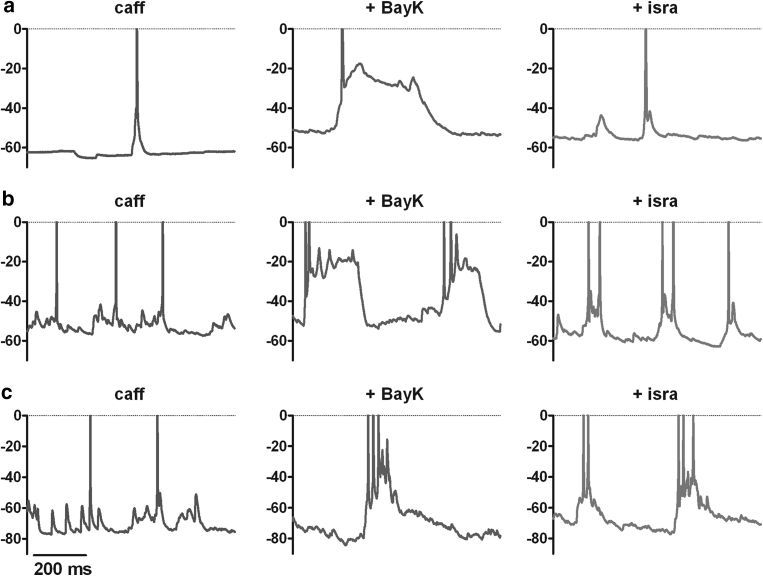
Diversity of BayK-induced PDS. Exemplary recordings from three neurons (**a**–**c**) show that when BayK is co-administered with caffeine (which on its own did not induce abnormal discharges, *left traces*) PDS of various shapes emerge (*middle traces*). Exchange of BayK for isradipine eliminates PDS (**a**), largely reduces (**b**) or fails to suppress these abnormal electrical events (**c**) (*right traces*)

Examples from this set of experiments are given in Fig. 5, which illustrates that PDS induction by BayK can be reversed fully (Fig. 5a), partially (Fig. 5b) or may be largely resistant to block of LTCCs with isradipine (Fig. 5c). It also shows that some variability exist among BayK-induced PDS, for example in the number of spikes and/or in the oscillatory activity riding on the depolarization wave. Yet abnormally high depolarization waves and concomitant decreasing spike firing activity characterized all of these PDS events.

### LTCC-dependent Induction of PDS by Oxidative Stress

**Fig. 6:**
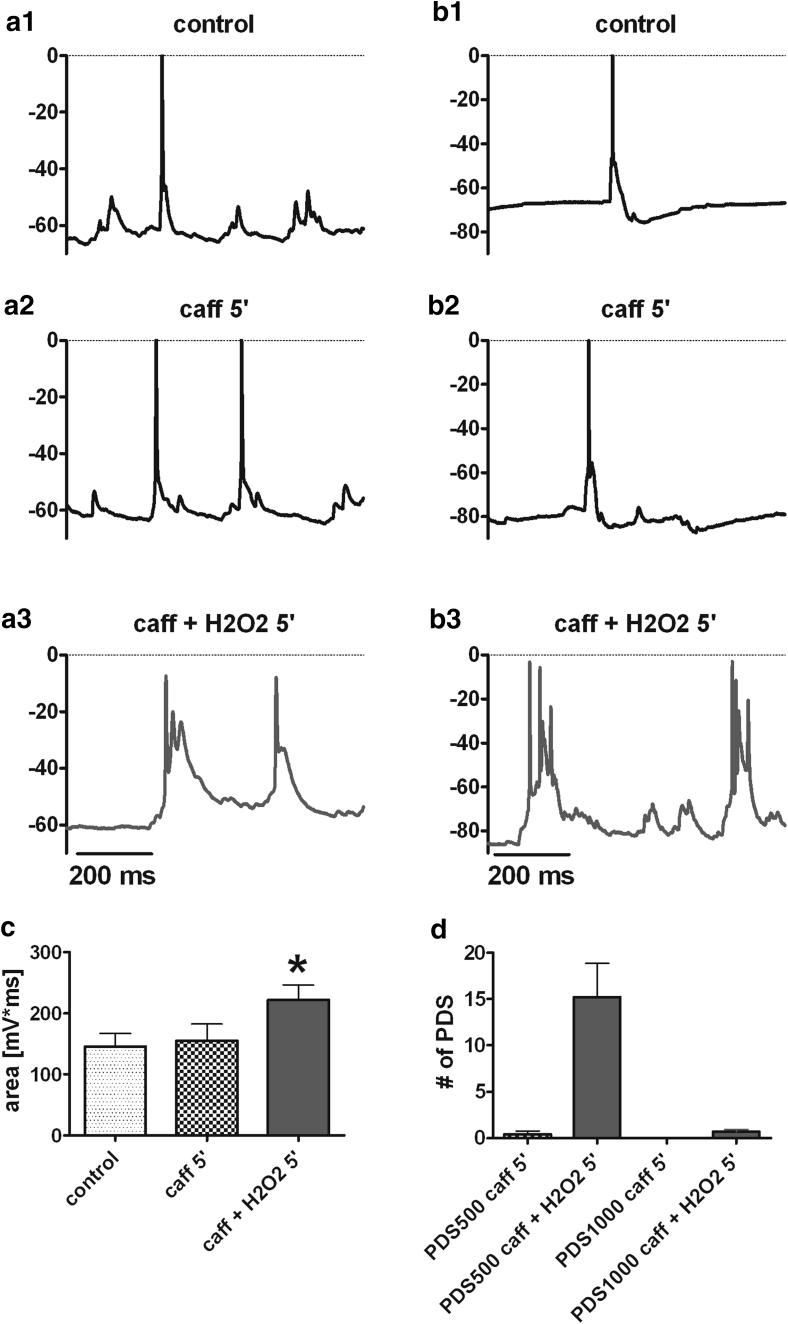
PDS induction by H_2_O_2_ in the presence of caffeine. Two examples of 3 mM H_2_O_2_-induced PDS are shown in the traces in **a** and **b**. Area and PDS1000 analysis from a total of 9 experiments is illustrated in the graphs in **c** an **d**. No alteration in discharge patterns was observed during a 5-min application of caffeine (traces in **a**2 and **b**2), but depolarization shifts emerged during a subsequent application of hydrogen peroxide (H_2_O_2_, see traces in **a**3 and **b**3). **c** A significant change in event area was only determined in recordings made in the presence of caffeine + H_2_O_2_ (repeated measures ANOVA followed by Dunnett’s multiple comparison test, *P ≤ 0.05). **d** The *graph* illustrates that the increase in event area by H_2_O_2_ is due to the formation of a distinct number of moderately enhanced electrical events (PDS500) but only individual PDS1000 within the 2-min time frame

**Fig. 7:**
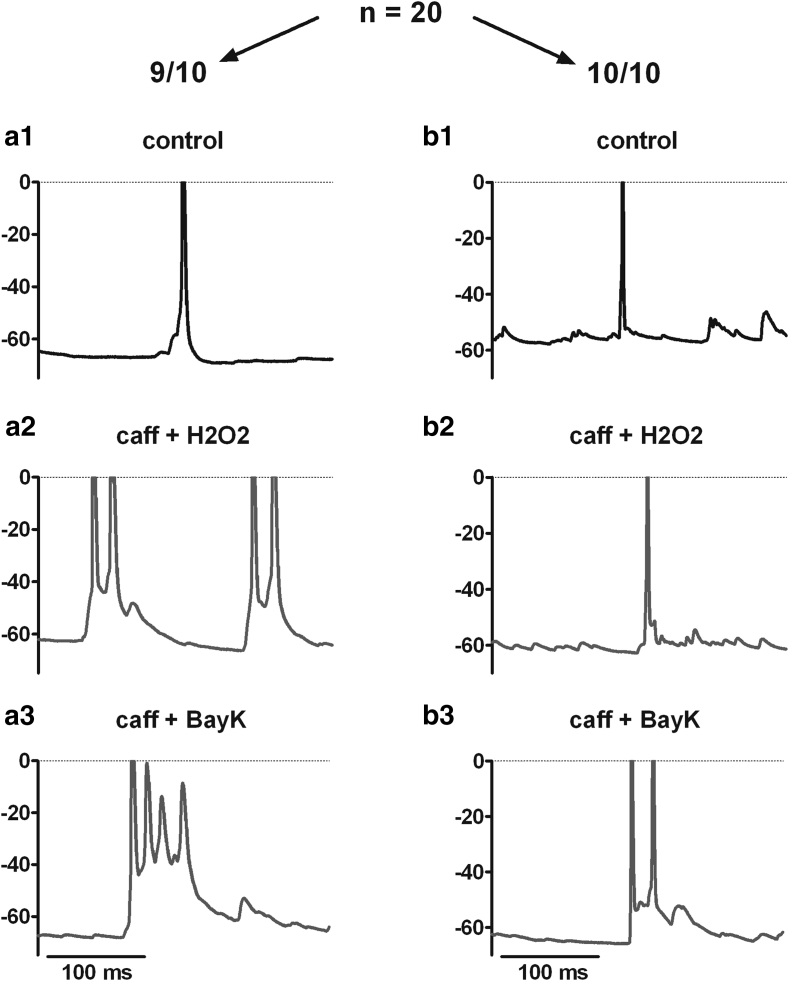
Induction of PDS with H_2_O_2_ requires LTCCs. As illustrated by original traces, 3 mM H_2_O_2_ only induced PDS in those of 20 neurons, where BayK also led to the appearance of depolarization shifts (*left column*, representative for 9 out of 10 cells in which BayK led to PDS formation, see *bottom trace*; in one cell with BayK-induced PDS, there was no effect with H_2_O_2_), but not in those which lacked a strong BayK-dependent effect (*right column*, representative for 10 out of 10 neurons, in which BayK only led to enhanced EPSPs at most, see *bottom trace*, **b**3)

**Fig. 8:**
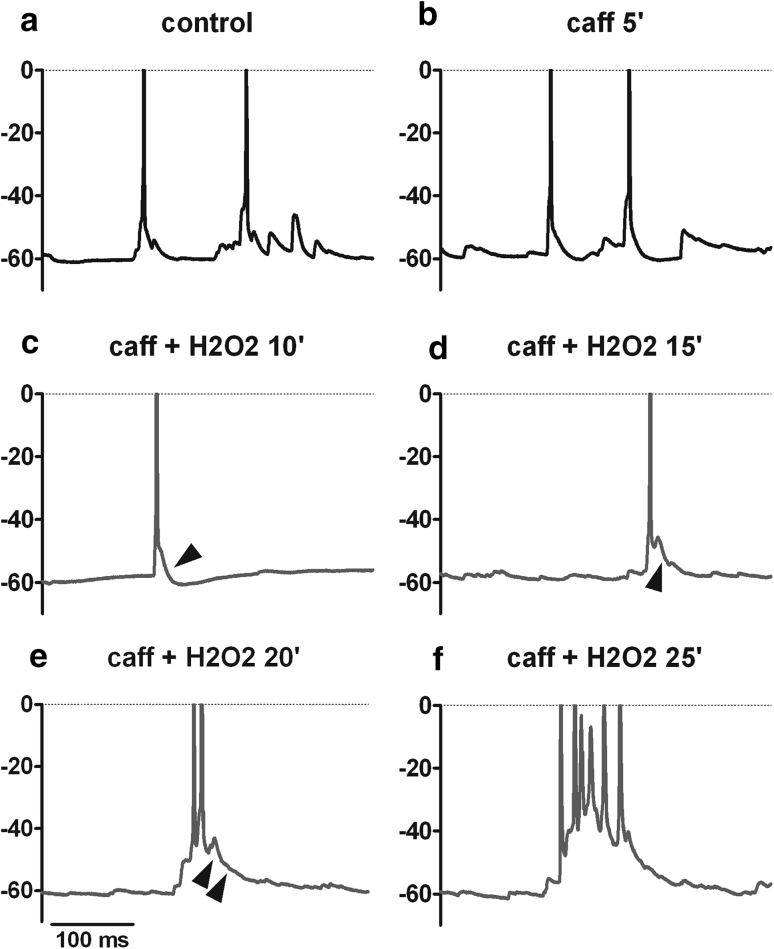
Low H_2_O_2_ concentrations slowly induce PDS formation. Example of a recording in which 100 μM H_2_O_2_ led to the delayed formation of PDS-like events. **a**–**c** Caffeine alone for 5 min (**b**) and in combination with H_2_O_2_ for further 10 min (**c**) did not affect discharge patterns, as compared to the control recording (**a**). **d**–**f** After about 15 min in caffeine + H_2_O_2_, enhancement of EPSPs occurred (showing up as a change in the spike after potential, see the *arrowheads* in **c** and **d**) which progressed (*arrowheads* in **e**) until PDS emerged, one is shown in **f**. Hence, augmentation of EPSPs (**d**, **e**) precedes the formation of PDS-like events (**f**)

So far, we had obtained evidence that PDS could be evoked by pharmacological potentiation of LTCCs. In the context of epilepsy (where PDS have been suggested to act in an epileptogenic manner, see for example Staley et al. 2005), we were interested whether the effects on LTCC activities by pathological means might also give rise to PDS. Enhancement of LTCC activity by H_2_O_2_ is a well-known effect, particularly in cardiac LTCCs (Thomas et al. 1998; Hudasek et al. 2004; Xie et al. 2009; Song et al. 2010) but has also been described for hippocampal LTCCs (Akaishi et al. 2004; Ishii et al. 2011). Mitochondrial dysfunction and oxidative stress have been suggested to represent a contributing link to acquired epilepsy. For example, increased H_2_O_2_ production in kainic acid- and lithium-pilocarpine-induced epileptogenesis animal models was seen in the “latent period,” that is where IIS/PDS also appear (Hellier et al. 1999; Waldbaum and Patel 2010). Similar to the results obtained with BayK in the caffeine assay of PDS formation, 1 mM caffeine alone was insufficient to evoke any PDS-like events. Yet upon administration of 3 mM H_2_O_2_, PDS-like events were discernible (*n* = 9, Fig. 6). However, H_2_O_2_-induced PDS-like events appeared less pronounced than those seen in the presence of BayK as evidenced from the event area analysis (Fig. 6c) and the number of PDS1000 induced (Fig. 6d, right bars). We additionally performed the determination of PDS500, and this analysis revealed clear evidence for a moderate PDS induction capability of hydrogen peroxide (Fig. 6d, left bars). Interestingly, H_2_0_2_ was only able to evoke PDS-like events in those neurons, where BayK administration had a distinct effect. This is shown in Fig. 7 where experiments are illustrated in which H_2_O_2_ was tested always prior to BayK (*n* = 20). In half of the neurons (10 out of 20), augmented depolarizing events appeared upon exchange of H_2_O_2_ for BayK (note that a similar percentage of neurons—6 out of 11—responded with PDS to BayK in the experiments presented in Fig. 3), and in 9 out of these 10 neurons, H_2_O_2_ had already enhanced depolarizing events (see the trace in A2 in Fig. 7). In contrast, H_2_O_2_ left neuronal activity entirely unaltered in the other 10 neurons, where subsequent application of BayK showed only a slight increase in EPSPs at most, as illustrated in Fig. 7b1–b3. This indicated that H_2_O_2_ only induced PDS-like events in neurons with a certain level of LTCC availability. To corroborate the finding that oxidative stress may contribute to the formation of PDS, we tested considerably lower concentrations of H_2_O_2_. As illustrated in Fig. 8 (the example shown is representative of three similar observations), PDS-like events also appeared upon administration of 100 μM hydrogen peroxide, but it took up to 30 min until events were induced that resembled PDS (Fig. 8f). Note that augmentation of EPSPs preceded the appearance of PDS-like events (Fig. 8d, e). The delayed induction of PDS-like events with 0.1 mM H_2_O_2_ was in contrast to the results obtained with 3 mM H_2_O_2_, which evoked such events typically within 5 min in responsive cells, although it left other electrophysiological parameters essentially unaffected in the non-responsive cells (hyperpolarization of the resting membrane potential in the range of a few millivolts or a somewhat enhanced action potential after-hyperpolarization was noted in some neurons, data not shown) even at these concentrations and within that time frame (3 mM H_2_O_2_ was tested for up to 10 min before BayK was applied at the end of the experiment, see Fig. 7).

### Differences in Proneness to PDS Formation

**Fig. 9:**
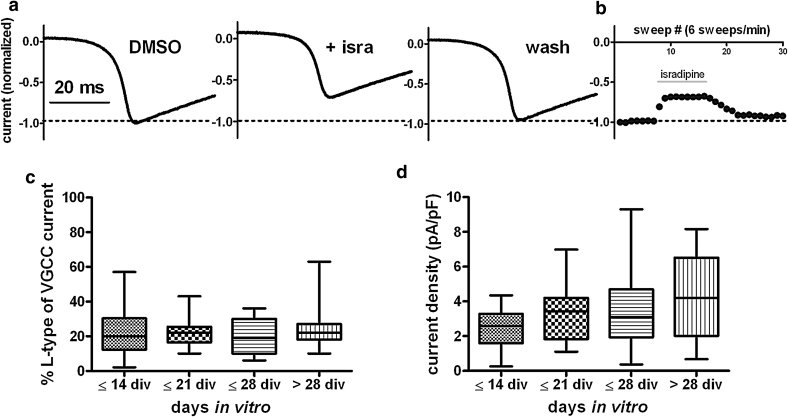
Levels of LTCC-mediated calcium currents in primary hippocampal neurons. **a** LTCC-mediated current components in total voltage-gated calcium currents were determined by applying ramp depolarizations (0.5 mV/ms) from −80 mV (=holding potential) to +50 mV and measurement of calcium current reduction upon a 90-s administration of 3 μM isradipine. The three traces depict the peak currents evoked under control conditions (DMSO), 3 μM isradipine and after washout of the dihydropyridine. **b** The reversible reduction was monitored by reading the peak of currents that were elicited every 10 s (e.g., sweeps 8–16 in the experiment shown). **c** Percentage of isradipine inhibited current with respect to total voltage-activated currents calculated from measurements as shown in **a**, **b**. Neurons were grouped according to the age of the cultures, as indicated on the *x-*axes. Neurons that had been kept in culture for at least 10 days but not longer than 2 weeks were allocated to the ≤14 days in vitro (DIV) group (*n* = 16), neurons that had been maintained in culture for more than 4 weeks and maximally up to 5 weeks were allocated to the >28 DIV group (*n* = 19). *n* for the ≤21 DIV and ≤28 DIV was 17 and 15, respectively. Considerably variation of LTCC current density exists in all age groups, yet statistically groups do not significantly differ from each other. **d** Same data as in c. LTCC current density (pA/pF) was determined by relating of the dihydropyridine-sensitive current component to cell capacitance as a measure of cell surface. To highlight the intrinsic variation, data in **c** and **d** are shown as box-plots with min to max whiskers

In qualitative terms, the effect of LTCC potentiation on brief excitatory events was unimodal both under otherwise untreated conditions and in caffeine-treated neurons. However, quantitatively considerable differences were observed (e.g., as depicted in Figs. 1, 3, 5). Hence, we wondered what the cause of this quantitative variability may be. We reasoned that a plausible explanation could lie in alternate endogenous LTCC activities. To address this possibility, we aimed to investigate the availability of LTCC channels by means of voltage-clamp recordings of calcium currents and determination of the percentage of LTCC currents in overall voltage-gated calcium currents and LTCC current densities (see “Materials and Methods” section for methodological details). By applying 260-ms-long voltage ramps from −80 mV (holding potential) to +50 mV (ramp speed 0.5 mV/ms), *U-*shaped inward currents were evoked, indicating that peak voltages of total calcium current activation were always reached. Hence, with the ramp protocol, it was possible to ensure maximal activation of voltage-gated calcium channel (VGCC) currents. While applying voltage ramps every 10 s, DMSO was applied, which at the concentrations tested (up to 0.3 %) did not affect the currents. We then added 3 μM isradipine and elicited currents until a stable reduction in the peak current was obtained or for a few minutes in cells with minor current changes. From the difference between the control peak current (determined in DMSO only) and the peak current measured in the presence of the LTCC inhibitor, we obtained an estimate of the current that was carried by LTCCs. An example of these experiments is illustrated in Fig. 9a, b, which shows a reversible reduction in the voltage-activated calcium current peak by isradipine of 28 %, thus representing an intermediate level of LTCC current contribution to VGCC currents within this age group (the age of the neuron was 20 days in vitro, and the range of current inhibition at this age was 10–44 %, mean 22.2 % ± 8.4 % standard deviation, see below). The high standard deviation was indicative of considerable variation. We wondered whether these differences were affected by the age in culture and therefore investigated neurons within a wide range of days in vitro (DIV). For statistical analysis, data were grouped according to age as indicated in Fig. 9c, d. It emerged that there was no statistical difference between LTCC current activities at various neuronal ages (this was true for both the data shown in Fig. 9c and in Fig. 9d), although the pronounced differences were present in all age groups: with respect to the percentage of dihydropyridine-sensitive current of total voltage-activated calcium current, it emerged that <10 % and up to ~60 % can be carried by L-type channels, depending on the neuron investigated. By relating the currents to cell capacitance (which was determined during the capacitance current compensation routine, see “Materials and Methods” section) LTCC current densities were calculated. As can be seen in Fig. 9d, this type of analysis yielded identical results: there is considerable variation in LTCC currents within hippocampal neurons (with densities covering a range from 0.25 to 9.3 pA/pF), but this variation was seen in all groups investigated and was thus independent of the time the neurons had been kept in culture.

### LTCC↑ Shows Bimodal Effects on Full-blown Seizure-like Activity

**Fig. 10:**
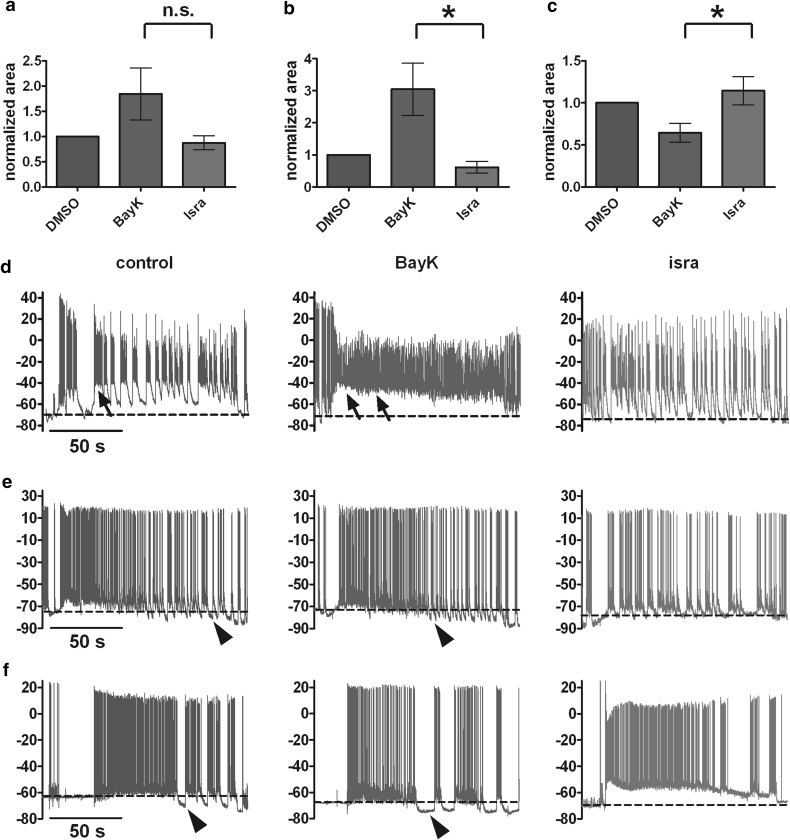
LTCCs can both enhance and reduce low-Mg^2+^-induced seizure-like activity. Seizure-like activity was induced repeatedly for 2-min with 5-min intervals, and DMSO, BayK or isradipine was co-administered in the low-Mg^2+^ saline. To account for differences in the delay till the onset, 90 s of SLA out of 120-s superfusion time were evaluated by determining the area between the trace and a baseline set at the membrane potential just prior to the change of the superfusion saline (dashed line in the original traces below). **a**–**c** The *graphs* show the results for all neurons (**a**, *n* = 14) and after separation of the data according to whether an increase (**b**, *n* = 7) or a decrease in the area (**c**, *n* = 7) was determined for SLA in BayK. Data are shown normalized to control areas (DMSO). Statistical difference between BayK and isradipine data was tested using Wilcoxon matched-pairs signed rank test (n.s. not significant; *P < 0.05). **d**–**f** Original traces illustrating the augmenting or reducing effect of LTCC modulation on SLA. The switch to low-Mg^2+^ saline was made within 15 s of the recordings shown, and the switch back to normal saline took place about 15 s before the end of the traces. In **d,** SLA is enhanced upon application of BayK in that a long-lasting up-state (*two arrows*) is induced, whereas brief up-states that where already present in the control recording (one marked with an *arrow*) appear shortened in the presence of isradipine (isra). **e**, **f** Two examples where SLA especially in the latter phase are accompanied between firing activity by hyperpolarization of the membrane potential (marked with *arrowheads*) below its initial resting level (*dashed line*), which appears earlier (**e**) and/or is more pronounced when BayK is present (**f**), and which is abolished in the presence of isradipine

Our data provided evidence that up-regulation of LTCCs enhanced EPSPs which under certain conditions, for example disturbed calcium homeostasis (caffeine experiments) or oxidative stress (hydrogen peroxide experiments), builds up to the formation of PDS. Hence, with respect to brief electrical events (on the time scale of up to several hundred milliseconds), the impact of enhanced LTCC activity appears unidirectional. This is in contrast to the bimodal effects we had observed in our previous study on longer-lasting depolarizations and discharge activities (see Fig. 6 in Geier et al. 2011). Therefore, we were wondering whether and in which manner potentiation of LTCCs would affect long-lasting seizure-like activity (SLA). To address this question, we employed the low Mg^2+^ model of epilepsy (see “Materials and Methods” section for experimental details). SLA was quantified by the determination of the area below the V_m_ trace within a 90-s time frame, starting at the onset of SLA (Fig. 10a–c). Because SLA typically comprises enhanced discharge activity as well as up-states (Fig. 10d–f), the area determined during the low-Mg^2+^ application period greatly exceeds the area during normal activity encountered in standard external buffer solution (not shown). The area measured for the second control SLA was used to normalize all values for statistical analysis. Comparing the recordings obtained under the three conditions from a total of 31 neurons, the following picture emerged: in 10 neurons, the change in area was not exceeding 10 % and these cells were thus assumed to lack significant LTCC-mediated contribution to SLA. In 7 further cells, a greater than 10 % reduction in area was obtained which was further decreasing upon subsequent addition of isradipine. These effects were thus considered as not related to LTCC activity (but probably due to SLA-induced progressive alterations), and the corresponding data were excluded from evaluation. Analysis of the data from the 14 remaining neurons is summarized in Fig. 10a. The bar graphs show that BayK led to an increase in the area by 1.84-fold on average, the increase being reversed upon administration of isradipine yielding an averaged area of 88 % of control. Yet, statistical analysis did not reveal a significant difference between areas determined in the presence of BayK and areas measured in the presence of isradipine (*P* value = 0.24, Wilcoxon matched-pairs signed rank test). However, closer inspection of the area data and the traces suggested that LTCC modulation led to opposing effects on SLA. In 7 neurons, BayK induced a clearly visible increase in activity, which was diminished when isradipine was applied, as illustrated in the example in Fig. 10d. In these neurons, the area increased by 1.3- to 7.0-fold, with an average of 3.0-fold. Upon exchange of BayK for isradipine SLA declined, then yielding a mean area of 61 % of control (Fig. 10b). In the 7 other neurons, the area decreased when BayK was administered (mean area 65 % of control) and increased on average 1.14-fold when isradipine was present (Fig. 10c). Illustrations of SLA recorded from neurons of this subgroup are given in Fig. 10e, f. For both effect modes, statistical analysis revealed significant differences between the areas recorded in BayK and isradipine (* in Fig. 10b, c indicates statistical significance with *P* values of 0.016 in both cases, Wilcoxon matched-pairs signed rank test).

Similarly, two opposing response modes were observed when abnormal discharge activity was evoked instead of low Mg^2+^ by application of a combination of two potassium channel inhibitors (XE/4AP), namely 10 μM XE-991 (M current inhibitor) and 100 μM 4-AP (A current inhibitor). Representative examples of recordings from a total of 34 neurons are depicted in an electronic supplementary figure (Online Resource 2). Again, the alteration in discharge activity obtained with BayK was reversed after exchange for isradipine. Together, these experiments reveal that potentiation of LTCCs can alter the severity of (long-lasting) SLA in opposing directions.

## Discussion

### LTCC↑ has the Capability to Evoke PDS

To investigate the implication of elevated LTCC activity in neuronal electrical excitation, the dihydropyridine-type agonist at LTCC channels BayK was used to potentiate channel activity. Pronounced effects of LTCC potentiation on EPSPs gave rise to events that were reminiscent of PDS, the cellular correlate of interictal spikes (Matsumoto and Ajmone Marsan 1964a; de Curtis and Avanzini 2001). This indicated a role of enhanced LTCC activity in the induction of these abnormal, potentially neuropathogenic electrical events. To test this possibility further, we employed caffeine because this agent was used in seminal in vitro studies on PDS formation (Moraidis et al. 1991). Thereby, PDS were evoked by BayK in 16 out of 27 neurons (Figs. 3, 4, 5). Hence, in the presence of caffeine, BayK led to PDS formation in about 60 % of the neurons. Re-evaluation of data we had obtained in the course of our previous study (Geier et al. 2011) revealed that without such pretreatment, BayK induced PDS in only less than 15 % of the neurons (data not shown).

In other words, although BayK can be envisaged to cause ubiquitous elevation of LTCC activity, only few neurons generated full-blown PDS as long as neuronal physiology was left otherwise experimentally unaltered. But under conditions of disturbed neuronal homeostasis (e.g., brought about by caffeine), PDS were evoked in a large subset of neurons. Hence, elevated activities of LTCCs render neurons prone to form pathological electrical events, but additional malfunctions (e.g., in intracellular calcium homeostasis) appear to be required for their actual occurrence. It should be noted that the disrupting stimuli exerted in our study (short-term exposure to caffeine, but also hydrogen peroxide) were on their own insufficient (caffeine) or entirely reliant on LTCC availability (H_2_O_2_, see Fig. 7) to alter neuronal functions in electrophysiological terms.

### Neurons Differ in Their Proneness to LTCC-dependent PDS Formation

The question why depolarization shifts arise in some neurons but not in others needs further consideration. The fact that small events remained unaltered by potentiation of LTCCs (see Fig. 2) suggests that effects on presynaptic transmitter release are not involved in the induction of PDS per se. Instead, PDS induction appears to be an effect relying on endogenous postsynaptic conductances that are activated by synaptic stimuli. LTCC-dependent depolarization shifts may involve coupling to Ca^2+^-dependent conductances. The main excitatory coupling in primary hippocampal neurons was identified by us recently to be mediated by activation of a Ca^2+^-dependent sodium conductance, for example non-selective cation channels (Geier et al. 2011). Unfortunately, the molecular nature of CAN channels remained unknown, and to date, no specific blocker of CAN channels is available. Hence, the question whether CAN channels contribute to PDS with an excitatory drive via cation influx cannot be answered at present. Arguing against such a possibility is a report by Schiller (2004), demonstrating that CAN channel activity does not play a prominent role in individual PDS but rather enables repetitive PDS discharge (runs of PDS). Alternatively, depolarization waves such as those seen in PDS may not necessarily require LTCC coupling. Ca_v_1.3 LTCCs, for example, have been suggested to carry window currents (e.g., Xu and Lipscombe 2001), so it is possible that continuous influx of Ca^2+^ via these channels directly contributes to the depolarization shift. Further research employing LTCC knockout mice (for example Cav1.3^−/−^ mice established by Platzer et al. 2000) or mice with disrupted TRPM channel expression (these channels are suspected to carry neuronal CAN channel currents, see for example Guinamard et al. 2011 or Mrejeru et al. 2011) may potentially be useful to address these hypotheses.

### Role of LTCC Density in the Inclination to PDS Formation

However, in this study, we moved on to explore mechanistic aspects of PDS induction in another direction. Augmentation of electrical events such as EPSPs by LTCC potentiation was also seen in those neurons not showing any PDS-like events (provided that the synaptic potentials exceeded the threshold for LTCC activation, whereas “small events” remained unaffected). This may be related to considerable variations in LTCC density among primary hippocampal neurons. Indeed, we obtained evidence for this possibility by determining isradipine-sensitive components of peak calcium currents measured in voltage-clamp recordings. As shown in Fig. 9, LTCC current densities covered a wide range, which was independent of the age of the neurons in culture. Hence, primary hippocampal neurons may have anything from low to high baseline LTCC availability. Observations made in the course of our previous study (Geier et al. 2011) on LTCC components of voltage responses to current injections also entirely support this notion (see Online Resource 3, which also addresses the difficulties of measuring LTCC currents in fully differentiated hippocampal neurons in perforated patch mode). Hence, differences in endogenous LTCC levels may explain the apparent continuum in the BayK-induced effects, ranging from a moderate enhancement of spontaneous depolarizing synaptic potentials to the formation of full-blown depolarization shifts.

### Pathogenetic Aspects of LTCC-dependent PDS

Elevated levels of LTCC activity were reported to occur for example in aged neurons, in neurons of epilepsy-prone animals and in oxidatively stressed neurons (Amano et al. 2001a, b; Thibault et al. 2001; Green et al. 2002; Veng and Browning 2002; Davare and Hell 2003; Park et al. 2003; Veng et al. 2003; Akaishi et al. 2004; Kang et al. 2004). Indeed, our experiments with hydrogen peroxide point to the possibility that oxidative stress may lead to PDS formation pathologically.

Although we sampled our data from all types of hippocampal neurons (see the addendum to the heterogeneity aspect in the electronic supplementary material, Online Resource 4), the effect of LTCC potentiation on synaptically induced short events was uniform in qualitative terms. Nevertheless, we noted some variation among the experimentally evoked PDS, irrespective of whether they were induced by BayK or H_2_O_2_. But this was not unexpected because similar observations have already been made in vivo in the first reports on these epileptiform events (Matsumoto and Ajmone Marsan 1964a, c).

The potential to induce PDS was generally smaller with H_2_O_2_ than with BayK. Yet pathologically, the less pronounced PDS-like events may be of higher relevance: it should be noted that epileptogenesis takes place over long time courses (e.g., weeks to months in animal models, see for example Morimoto et al. 2004 or Williams et al. 2009) and can thus be envisaged to be driven by events such as those induced in the course of oxidative stress rather than by events evoked with BayK. The latter appeared to lead to persistent changes in discharge patterns already within the time frame of our experiments (Fig. 4), which is of interest mechanistically but obviously does not fit into epileptogenic time scales seen in vivo (Dudek and Staley 2011). The irreversibility of strong PDS induction may be related to persistent structural or functional changes induced by pulsative Ca^2+^ rises that were shown to go along with PDS occurrence (Amano et al. 2001b; Schiller 2004). Such changes in neuronal excitability may no longer be maintained by LTCC activity alone. Obviously, this possibility needs further investigations that lie far beyond the scope of the present study. In fact, experiments to address this question are not trivial but certainly worth of future considerations since they touch closely on the proposed pro-epileptic potential of PDS.

### Opposing Effects of LTCC↑ on Disfunctional Neuronal Discharge Activities

In contrast to the unimodal situation with PDS, experiments on low-Mg^2+^ and XE/4AP-induced SLA, respectively, showed that potentiation of LTCCs can alter abnormal discharge activity in opposing manners, leading to enhancement involving plateau potentials on the one hand and reduction involving more pronounced after-hyperpolarizations on the other hand. This ambivalence was not unexpected because of the divergent effects of LTCC activation that we had found earlier for current-induced depolarizations of these neurons (Geier et al. 2011). Importantly, SLA, despite some degree of modulation, could be evoked under all conditions of LTCC modulation, namely under normal levels of LTCC activities (control recordings in the presence of vehicle), when LTCC activities were potentiated (BayK) and in particular when LTCC activity was blocked (isradipine).

## Conclusion

Taken together, this study provides evidence that the bimodal effects of LTCC activation on normal excitability shown earlier (Geier et al. 2011) can be extended to abnormal neuronal discharge activity. Our earlier study also demonstrated that bimodal LTCC coupling was only relevant at more long-lasting depolarizations (e.g., exceeding 0.5–1 s), whereas shorter depolarizations were unequivocally enhanced by LTCC activity [as can be seen in supplementary recordings made in the presence of TTX (e.g., Figure B in Online Resource 3), early on during long-lasting depolarizations—for example within the first second—LTCC activity has enhancing effects (depolarizations exceed the traces recorded in the presence of isradipine!), irrespective of the subsequent excitatory or inhibitory LTCC-mediated outcome]. We extended this finding in the present study showing that enhanced activity of LTCCs augments EPSPs and eventually gives rise to PDS in susceptible cells. Notably, no inhibitory effect of LTCC potentiation was observed on short depolarizing events. This is in contrast to the situation with long-lasting abnormal discharge activity. Our data on SLA suggest that therapeutic reduction in LTCC activity may have little beneficial or even adverse effects on epileptic seizures, which may help to explain the opposing effects of LTCC inhibition on seizures seen in clinical trials (Kulak et al. 2004). However, because evidence is continuously accumulating that PDS represent important elements in epileptogenesis (Dyhrfjeld-Johnsen et al. 2010; Staley et al. 2011), LTCCs may provide valuable targets for anti-epileptogenic rather than anti-epileptic therapy. Moreover, interictal spikes have besides epileptogenesis also been implicated in other neurologic disorders, such as attention deficit disorder, anxiety disorders and psychoses (for a review see Barkmeier and Loeb 2009). Hence, new therapeutic strategies to counteract PDS may serve in the therapeutic prophylaxis of acquired epilepsies but could also be of value in other neuropathologies.

## Electronic supplementary material

Below is the link to the electronic supplementary material.

Supplementary material 1 (DOC 466 kb)

Supplementary material 2 (DOC 1623 kb)

Supplementary material 3 (DOC 1320 kb)

Supplementary material 4 (DOC 31 kb)
