# Supplementary material for: Raised Activity of L-Type Calcium Channels Renders Neurons Prone to Form Paroxysmal Depolarization Shifts
Source: Neuromolecular Med. 2013 May 22;15(3):476–92. doi: 10.1007/s12017-013-8234-1 (PMC3732764; doi:10.1007/s12017-013-8234-1)
Supplement: Supplementary file 1 — Supplementary material 1 (DOC 466 kb) [file 12017_2013_8234_MOESM1_ESM.doc]

**Online Resource 1: Details on event detection and quantification.**


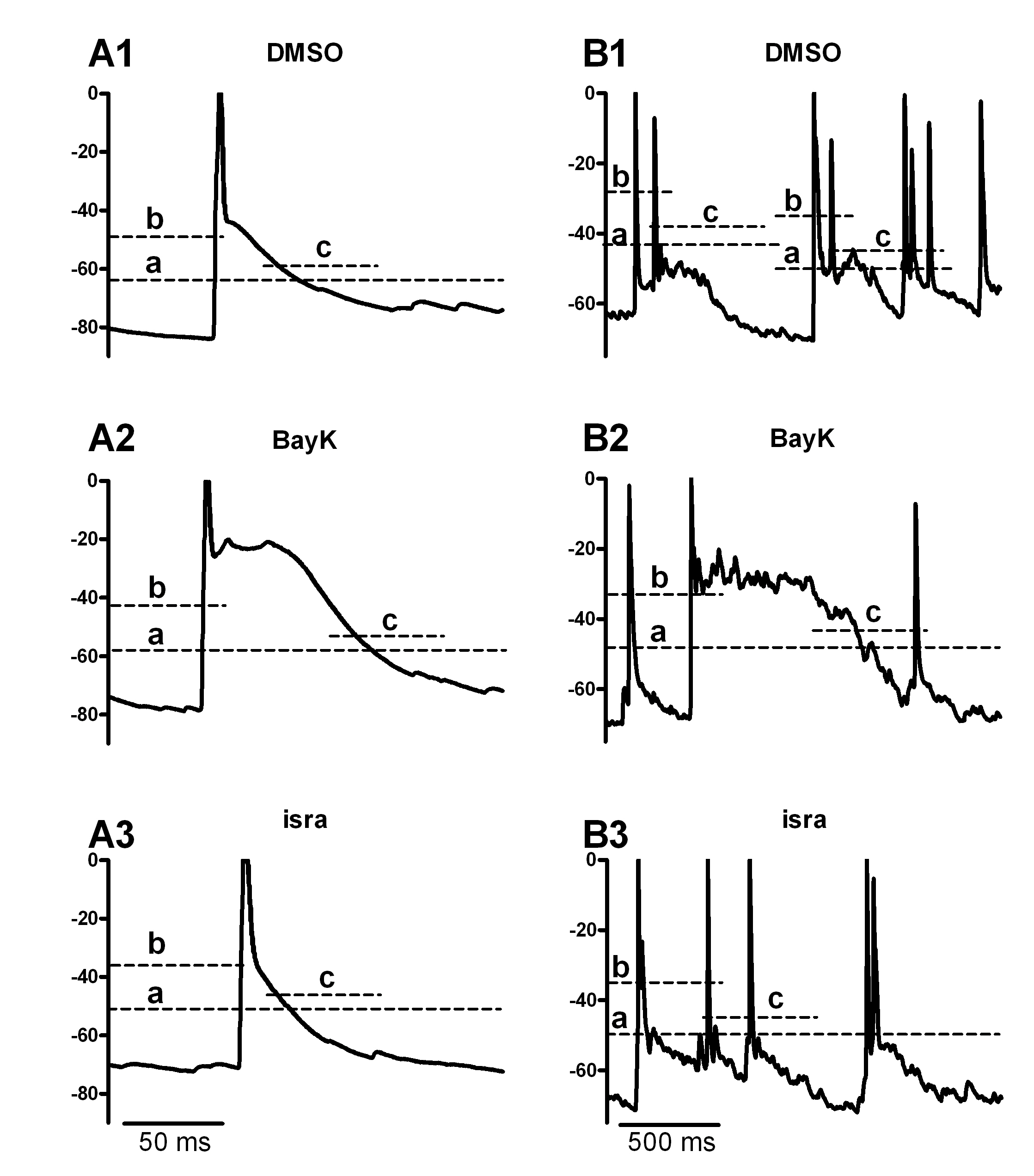


**Figure OR1: Event detection routines used for identification and quantification of depolarization shifts in current clamp recordings of perforated-patch primary hippocampal neurons using threshold based searches.** The original traces show two examples (A, B) of depolarization shifts appearing upon application of BayK. Y-axes units are in mV. For the analysis of these electrical events a threshold search operation („event detection“) was performed using Clampfit 10.2, which is part of the pCLAMP 10 electrophysiology data acquisition and analysis software package (Molecular Devices, Sunnyvale, CA, USA). The dashed horizontal lines in the control (DMSO) recordings (A1, B1), in the recordings made upon application of BayK (A2, B2) and in the recordings made after exchange of BayK for isradipine (A3, B3) indicate (a) a virtual baseline set 20 mV above the membrane resting potential, (b) the threshold set at 15 mV above the virtual baseline and (c) the re-arm potential set at 5 mV above the virtual baseline. Noise rejection was set to 1 ms. Using these parameters only supra-threshold events are identified and event area is determined as the area between the trace and the virtual baseline (a) starting at the point where the voltage exceeded the threshold (b, 35 mV above the actual baseline) and ending where it undershoots the virtual baseline (a), or when the threshold (b) is again exceeded from a voltage below the re-arm value (c). This procedure enabled us to specifically target the analysis to pronounced levels of depolarization rather than to - for example - depolarizing envelops which would also be present in burst firing, or long-lasting EPSPs.
