# Supplementary material for: Raised Activity of L-Type Calcium Channels Renders Neurons Prone to Form Paroxysmal Depolarization Shifts
Source: Neuromolecular Med. 2013 May 22;15(3):476–92. doi: 10.1007/s12017-013-8234-1 (PMC3732764; doi:10.1007/s12017-013-8234-1)
Supplement: Supplementary file 2 — Supplementary material 2 (DOC 1623 kb) [file 12017_2013_8234_MOESM2_ESM.doc]

**Online Resource 2: Effect of LTCC modulation demonstrated on an alternative experimental model of epileptiform activity.**


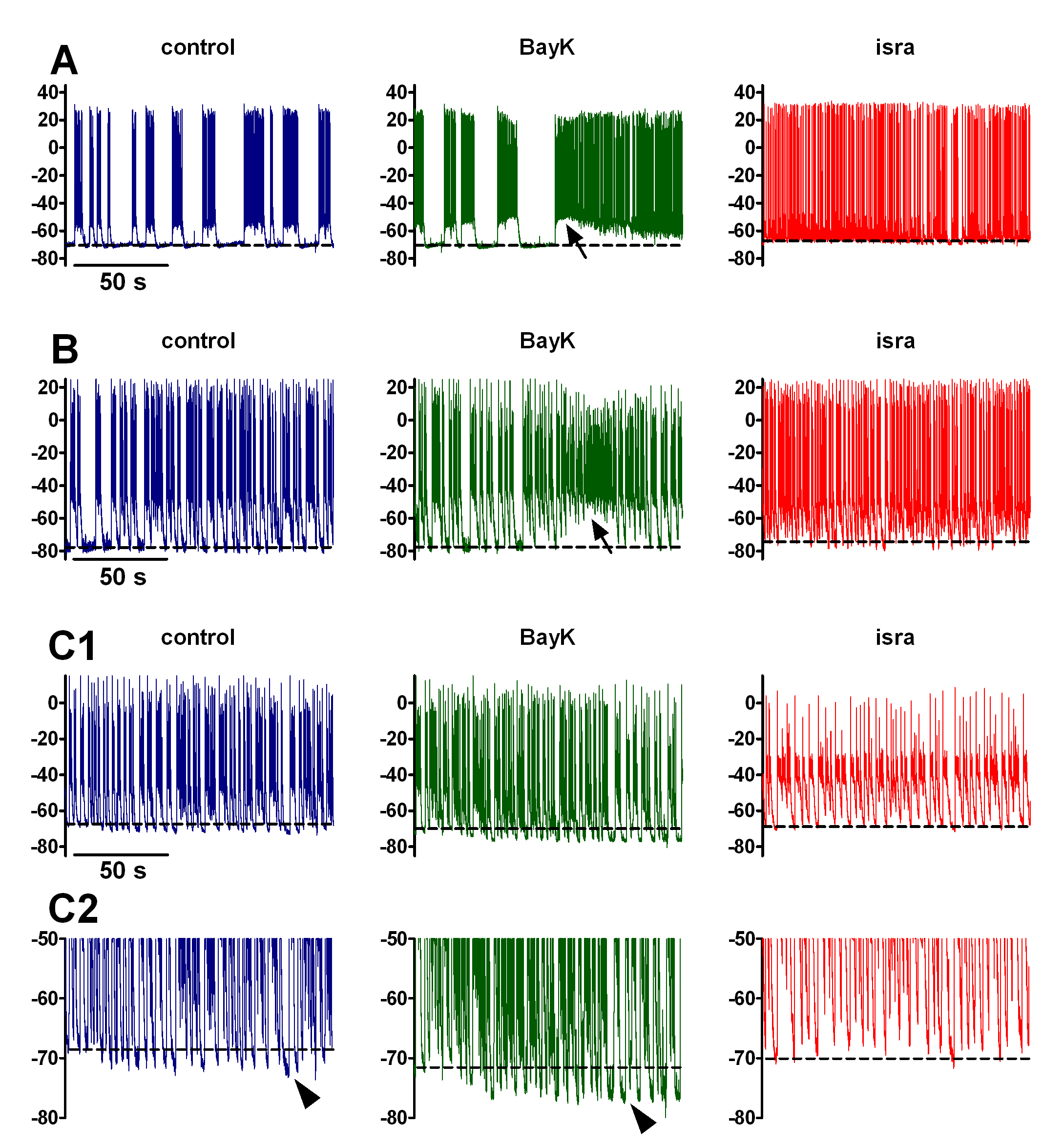


**Figure OR2: LTCCs can both enhance and reduce XE/4AP induced seizure-like activity.**

As an alternative *in vitro* model of seizure-like activity, co-application of the potassium channel inhibitors XE-991 and 4-aminopyridine in standard external buffer (XE/4AP) was used [mutations in both M-type (K_v_7 family) and A-type potassium channels (e.g. K_v_4.2 channels) are known to cause epileptic syndromes (Fransen and Tigerholm 2010; Maljevic et al. 2010; Singh et al. 2006)]. The figure depicts three representative examples of the effect of LTCC modulation on abnormal neuronal discharge activity induced by XE/4AP (n=34). Note that the control recordings in the left column show the neuronal activity after addition of the potassium channel inhibitors, which intensified the discharge pattern as compared to the respective neuronal activity in drug-free solution (not shown). For the investigation of LTCC-mediated effects, recordings of XE/4AP-induced abnormal electrical activity where made when vehicle (DMSO) was present alone (=control), or in the presence of BayK and isradipine. **A**, **B** In the examples shown discharge activity is augmented upon administration of BayK which can be seen in the selected parts of the recordings as a long lasting up-states (arrows), whereas only brief up-states are present in the control recording, and up-states are entirely absent (A) or are reduced (B) in the trace recorded in the presence of isradipine. **C** Example showing the opposite effect with up-states in the control recording and even more so in the recording made in the presence of isradipine, whereas upon administration of BayK up-states are reduced with a concomitant appearance of after-hyperpolarizations (C1). The dependence of after-hyperpolarizations (one each marked with an arrowhead in the control and in the BayK recording) on LTCC activity is illustrated in the magnification of recordings in the area of the resting membrane potential in C2, in that they are enhanced by BayK (as compared to control) and abolished by isradipine. The units on the Y-axis represent mV.

**References**

Fransen, E., Tigerholm, J. (2010). Role of A-type potassium currents in excitability, network synchronicity, and epilepsy. Hippocampus, 20(7), 877-887.

Maljevic, S., Wuttke, T. V., Seebohm, G., Lerche, H. (2010). KV7 channelopathies. Pflugers Archive, 460(2), 277-288.

Singh, B., Ogiwara, I., Kaneda, M., Tokonami, N., Mazaki, E., Baba, K., Matsuda, K., Inoue, Y., Yamakawa, K. (2006). A Kv4.2 truncation mutation in a patient with temporal lobe epilepsy. Neurobiology of Disease, 24(2), 245-253.
