# Supplementary material for: Raised Activity of L-Type Calcium Channels Renders Neurons Prone to Form Paroxysmal Depolarization Shifts
Source: Neuromolecular Med. 2013 May 22;15(3):476–92. doi: 10.1007/s12017-013-8234-1 (PMC3732764; doi:10.1007/s12017-013-8234-1)
Supplement: Supplementary file 3 — Supplementary material 3 (DOC 1320 kb) [file 12017_2013_8234_MOESM3_ESM.doc]

**Online Resource 3: Comment on and additional evidence for widely varying levels of endogenous LTCC availability in hippocampal neurons.**

The difficulties of measuring LTCC currents in fully differentiated hippocampal neurons have already been outlined in the results section of the main text. However, we would like to point out that overall, data obtained with our voltage clamp approach closely matched the results of other authors, e.g. with respect to the mean percentile contribution of LTCC current to total VGCC-mediated currents (see for example Avery and Johnston, 1996; Ishibashi et al., 1998, Blalock et al., 1999; Deak et al., 2000, Pravettoni et al., 2000). Our current densities are also in the same range as reported previously by others (see for example Blalock et al., 1999). Hence, we are positive that we have done everything that could possibly be done to get an idea of the levels of LTCC availability in the primary hippocampal neurons. Moreover, with respect to variability of LTCC availability among hippocampal neurons, voltage clamp and current clamp experiments qualitatively yielded the same result: our previous work (on 84 neurons in total) showed that the type of the LTCC-mediated response can be used as a read out of the availability of LTCC channels (Geier et al. 2011). With moderate activation the excitatory response mode prevailed, which turned into oscillatory activity and then gave way to counteraction of excitation at the more pronounced levels of LTCC activation, particularly in the presence of BayK (as it is the case for the neuron from which recordings shown in Figure OR3A were made). This response pattern could be observed in many hippocampal neurons, although some neurons showed a clear deviation in that they lacked any signs of LTCC-mediated responses or showed hyperpolarizing sags already under endogenous, unpotentiated levels of LTCC activity: see the figures below contrasting (Figure OR3A) the widely seen transition-trend from excitatory via oscillatory to inhibitory LTCC-mediated voltage responses as the level of depolarization and Ca^2+^ availability (presence of BayK, right column) increases, with (Figure OR3B) recordings from neurons that deviated from the typical trend , because they continued to lack at the most pronounced levels of depolarization any signs of LTCC activity or showed a sag like response already prior to the application of BayK. Hence, some of the neurons appear to have very little LTCC availability, whereas in others endogenous LTCC activity is high enough to enable “hyperpolarizing sag” responses already under control conditions. These observations indicate that voltage clamp and current clamp experiments qualitatively yield the same result with regard to differences in endogenous LTCC activity levels. Hence, we are confident that the problems inherent to the former voltage-clamp approach, in particular in the case of perforated patch recordings, are negligible, at least with respect to the question that these experiments were intended to answer (namely to test if and to what extent LTCC availability may differ within the neurons investigated).

In conclusion, considerable variability in LTCC levels (e.g. current density and percentage of total voltage-gated calcium channel-mediated currents, respectively) was observed within age groups, whereas the mean LTCC levels were not significantly different between age groups. Variability in PDS formation was also found within age groups, not between age groups. This is entirely in favor of the idea that Ca^2+^-entry is the determining factor for PDS variability and is in line with the finding that PDS formation is related to potentiation of L-type channels.


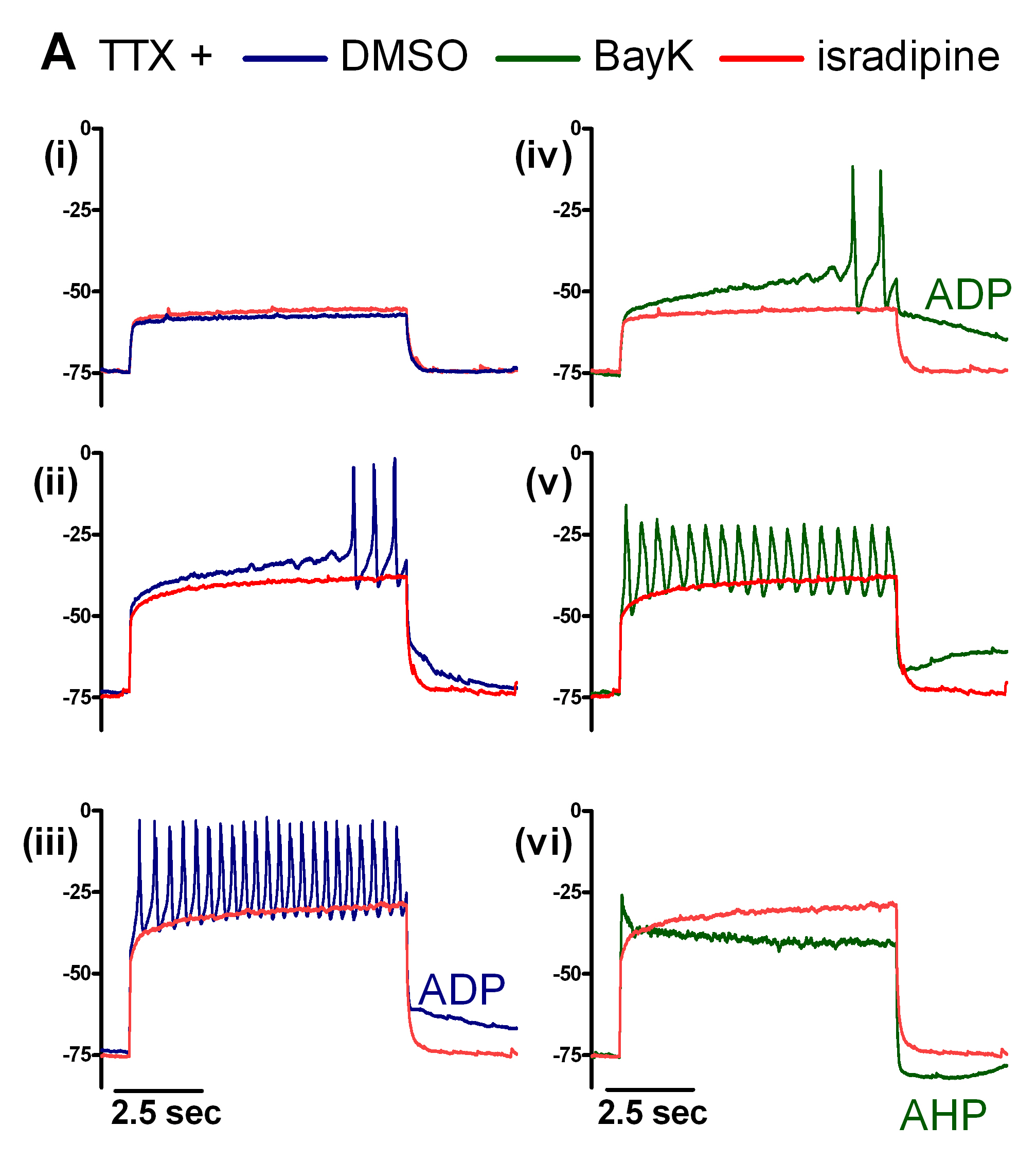


**Figure OR3A**. Recordings illustrating the typical transition from excitatory (“bump”), via oscillatory to inhibitory (“hyperpolarizing sag”) LTCC-mediated response modes as the level of experimentally induced depolarization was increased. Y-axes units are in mV. For methodological details see Geier et al. 2011. In brief current injections were increased stepwise to depolarize the neurons to various voltages between the resting membrane potential and about -25 mV. This was done in the presence of TTX and isradipine, conditions under which voltage responses remained largely passive (red traces). The same current injections were also applied when DMSO [dark blue traces, left column (i) to (iii)] or BayK [green traces, right column (iv) to (vi)] was present instead of isradipine, to enable activation of unpotentiated (DMSO) and potentiated LTCC channels (BayK), respectively. The overlay of recordings made in the presence of isradipine with recordings made when LTCC channels were unblocked shows that excitatory (“bump”) responses that are accompanied by after-depolarizations (ADP) are evoked first [(ii), (iv)], whereas in the course of more pronounced depolarizations oscillatory activity develops [at later stages in (ii) and (iv), and throughout the stimulation in (iii) and (v)]. Increasing the depolarizations further leads to reduction of the oscillation amplitudes and finally (especially in the presence of BayK) to the appearance of a hyperpolarizing component in the voltage response (“hyperpolarizing sag”) that is typically associated with after-hyperpolarizations (AHP) [see (vi)]. Note that all the responses shown were recorded from the same neuron, with the same incremental current injections under all three conditions. Moreover, recordings shown in the same row were evoked by identical current injections. Our earlier study (Geier et al. 2011) has shown that alternate coupling to excitatory or inhibitory Ca^2+^-dependent conductances is responsible for these distinct response modes. Moreover, we provided evidence that the response mode depends on the level of LTCC-mediated Ca^2+^ influx. In line with that notion, “hyperpolarizing sag”-responses were rarely seen in the absence of BayK, but were frequently observed when the LTCC agonist was present [e.g. as shown in (vi)].

**Figure OR3B** (below). In the absence of LTCC potentiation, bump and oscillatory activity - depicted in (i) and (ii), respectively - were the most common forms of LTCC-mediated responses visible in depolarizations to ≥ -30 mV. However, we also identified neurons that generated no LTCC-mediated response (iii) - not even after addition of BayK (not shown) - or generated a hyperpolarizing sag already under conditions of pharmacologically unaltered LTCC activity (iv), even though this response mode was typically seen only after application of BayK and with the most pronounced levels of depolarization [see Figure OR3A, (vi)]. The units on the Y-axis represent mV.


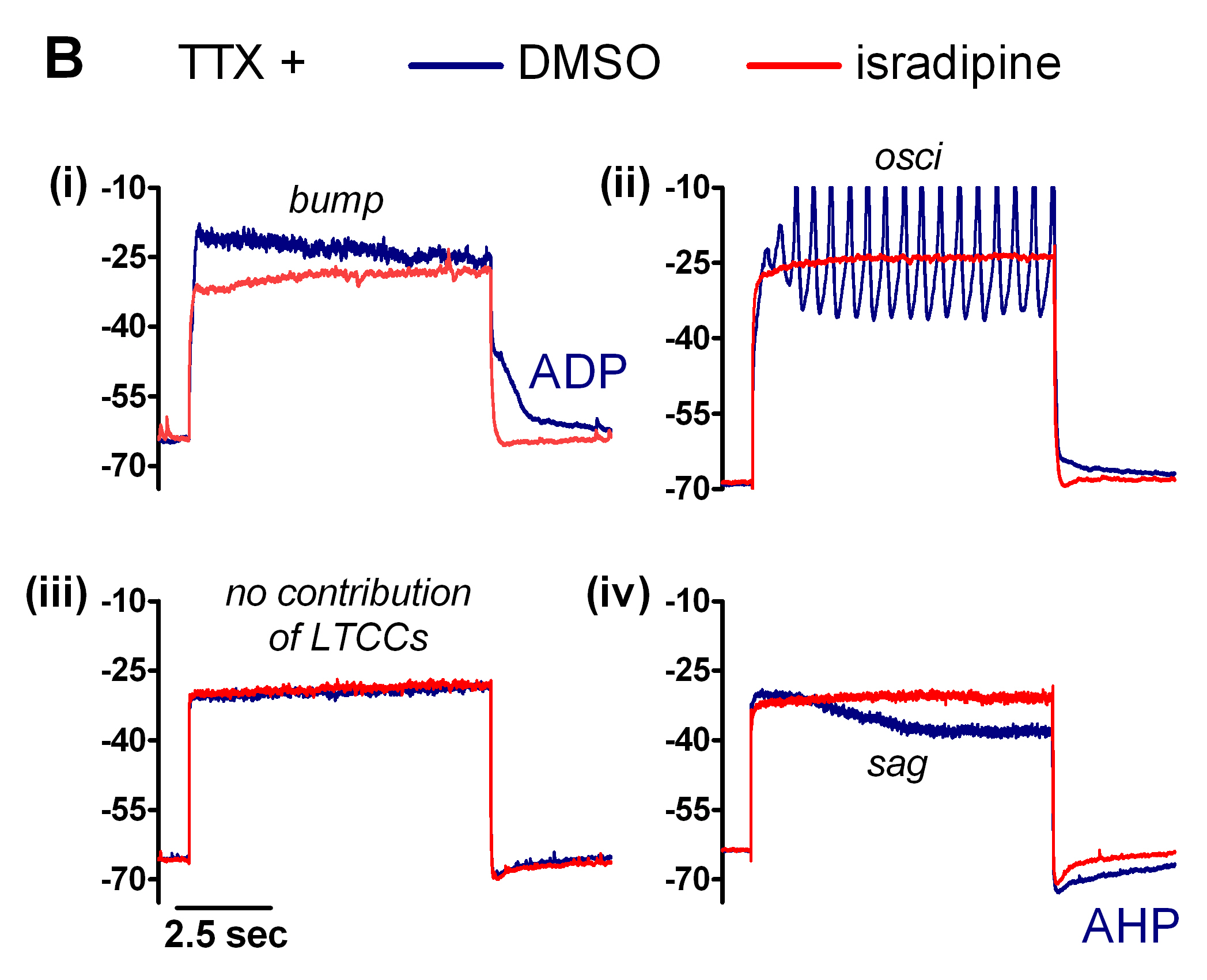


**References**

Avery, R. B., Johnston, D. (1996). Multiple channel types contribute to the low-voltage-activated calcium current in hippocampal CA3 pyramidal neurons. Journal of Neuroscience, 16(18), 5567-5582.

Blalock, E. M., Porter, N. M., Landfield, P. W. (1999). Decreased G-protein-mediated regulation and shift in calcium channel types with age in hippocampal cultures. Journal of Neuroscience, 19(19), 8674-8684.

Deak, F., Lasztóczi, B., Pacher, P., Petheö, G. L., Valéria Kecskeméti, Spät, A. (2000). Inhibition of voltage-gated calcium channels by fluoxetine in rat hippocampal pyramidal cells. Neuropharmacology, 39(6), 1029-1036.

Geier, P., Lagler, M., Boehm, S., Kubista, H. (2011). Dynamic interplay of excitatory and inhibitory coupling modes of neuronal L-type calcium channels. American Journal of Physiology-Cell Physiology, 300(4), C937-C949.

Ishibashi, H., Murai, Y., Akaike, N. (1998). Effect of nilvadipine on the voltage-dependent Ca^2+^ channels in rat hippocampal CA1 pyramidal neurons. Brain Research, 813(1), 121-127.

Pravettoni, E., Bacci, A., Coco, S., Forbicini, P., Matteoli, M., Verderio, C. (2000). Different localizations and functions of L-type and N-type calcium channels during development of hippocampal neurons. Developmental Biology, 227(2), 581-594.
