# Supplementary material for: Raised Activity of L-Type Calcium Channels Renders Neurons Prone to Form Paroxysmal Depolarization Shifts
Source: Neuromolecular Med. 2013 May 22;15(3):476–92. doi: 10.1007/s12017-013-8234-1 (PMC3732764; doi:10.1007/s12017-013-8234-1)
Supplement: Supplementary file 4 — Supplementary material 4 (DOC 31 kb) [file 12017_2013_8234_MOESM4_ESM.doc]

**Online Resource 4: Addendum on benefits and possible limitations of experiments on hippocampal neurons in primary culture.**

Primary hippocampal neurons are a well established experimental model in particular for *in-vitro* epilepsy research (see for example papers by the group of Robert DeLorenzo, e.g. Blair et al., 2008, or papers by Michael Segal, e.g. Segal, 1994) and provide disadvantages but also advantages over acute slice preparations. An obvious advantage of primary hippocampal neurons over slice preparations lies in the fact that in the cultures neurons are allowed to recover from stress conditions that are unavoidable in acute preparations. Primary hippocampal cultures can be maintained successfully for very long periods, e. g. 90 days by Segal and Furshpan (1990). Furthermore, drugs can be applied in defined concentrations directly onto neurons under investigations, without the need to allow for diffusion of the compounds through cell layers. Hence concentrations can be kept low, thereby warranting pharmacological selectivity. An obvious disadvantage of primary cultures lies in the heterogeneity of the neurons, whereby the origin of the neurons with respect to hippocampal subfields cannot be retraced. Hence, data are sampled both from pyramidal and granule cells, as well as from various types of interneurons.

On the other hand, this later feature of primary cultures can also provide an advantageous situation: effects that occur in cells of various morphological phenotypes can be assumed to be independent of the actual functional identity of the hippocampal neurons. For example, in our previous study on primary hippocampal neurons (Geier et al., 2011), only three major LTCC-mediated response modes could be exemplified, and we found that all three can occur in a single neuron (albeit at different levels of depolarization). We identified these responses in large number of cells, irrespective of other peculiarities that may be related to the specificity of the cell types. Hence, the acute effects of LTCCs in electrical events appear to be similar throughout different hippocampal neurons. Probably general somatodendritic mechanisms are more relevant for the role of LTCC in excitability than the cell shape or presynaptic mechanisms, e.g. type of neurotransmitter released at the terminals.

However, another concern when working with primary neurons relates to the possibility of cell identity loss when cultures are maintained for longer periods of time. Unfortunately, the degree of such loss of cell identity has not been addressed adequately so far. Having said that it is also worth mentioning that the preparation that we used in the current study has been employed successfully in a series of publications reporting on hippocampal neurophysiology (see papers published by Stefan Boehm’s group (a co-author of this manuscript), e.g. Boehm and Betz, 1997; Boehm, 1999; O’Connor et al., 1999; Goetze et al., 2006; Dorostkar and Boehm, 2007). Additionally, we found in the course of this study that LTCC current density did not change and that the contribution of LTCC currents to overall VGCC currents remained unaltered within the culture period (22.6 ± 3.5 % before 14 days in vitro, 26.1 ± 3.1 % after 4 weeks). What is more, RT-PCR determination of relative expression levels of neuronal LTCCs showed no difference between RNA samples taken from intact hippocampus and from cultured hippocampal neurons (unpublished data obtained in collaboration with G. Obermair, University of Innsbruck, who published corresponding data on mouse hippocampal neurons, see Schlick et al., 2010). Further more, we obtained evidence in previous work (Geier et al., 2011) that dissociated hippocampal neurons maintain hippocampal neuron characteristics even after long-term culture (e.g. LTCC-SK coupling as opposed to LTCC-BK coupling present in other central neurons).

Hence, while some limitations exist, our experimental approach employing primary hippocampal neurons allowed us to perform our investigations not on a narrowly defined type of neuron (e.g. CA1 neuron) but on a representative network of central neurons.

**References**

Blair, R. E., Sombati, S., Churn, S. B., Delorenzo, R. J. (2008). Epileptogenesis causes an N-methyl-d-aspartate receptor/Ca^2+^-dependent decrease in Ca^2+^/calmodulin-dependent protein kinase II activity in a hippocampal neuronal culture model of spontaneous recurrent epileptiform discharges. European Journal of Pharmacology, 588(1), 64-71.

Boehm S. (1999). Presynaptic alpha2-adrenoceptors control excitatory, but not inhibitory, transmission at rat hippocampal synapses. Journal of Physiology, 519(Pt 2), 439-449.

Boehm, S., Betz, H. (1997). Somatostatin inhibits excitatory transmission at rat hippocampal synapses via presynaptic receptors. Journal of Neuroscience, 17(11), 4066-4075.

Dorostkar, M. M., Boehm, S. (2007). Opposite effects of presynaptic 5-HT3 receptor activation on spontaneous and action potential-evoked GABA release at hippocampal synapses. Journal of Neurochemistry, 100(2), 395-405.

Geier, P., Lagler, M., Boehm, S., Kubista, H. (2011). Dynamic interplay of excitatory and inhibitory coupling modes of neuronal L-type calcium channels. American Journal of Physiology-Cell Physiology, 300(4), C937-C949.

Goetze, B., Tuebing, F., Xie, Y., Dorostkar, M. M., Thomas, S., Pehl, U., et al. (2006). The brain-specific double-stranded RNA-binding protein Staufen2 is required for dendritic spine morphogenesis. Journal of Cell Biology 172(2), 221-231.

O'Connor, V., El Far, O., Bofill-Cardona, E., Nanoff, C., Freissmuth, M., Karschin, A., et al. (1999). Calmodulin dependence of presynaptic metabotropic glutamate receptor signaling. Science, 286(5442), 1180-1184.

Schlick, B., Flucher, B. E., Obermair, G. J. (2010). Voltage-activated calcium channel expression profiles in mouse brain and cultured hippocampal neurons. Neuroscience, 167(3), 786-798.

Segal, M. M., Furshpan, E. J. (1990). Epileptiform activity in microcultures containing small numbers of hippocampal neurons. Journal of Neurophysiology, 64(5), 1390-1399.

Segal MM. Endogenous bursts underlie seizurelike activity in solitary excitatory hippocampal neurons in microcultures. J Neurophysiol. 1994 Oct;72(4):1874-84.
